# How one might miss early warning signals of critical transitions in time series data: A systematic study of two major currency pairs

**DOI:** 10.1371/journal.pone.0191439

**Published:** 2018-03-14

**Authors:** Haoyu Wen, Massimo Pica Ciamarra, Siew Ann Cheong

**Affiliations:** 1 Division of Physics and Applied Physics, School of Physical and Mathematical Sciences, Nanyang Technological University, Singapore, Singapore; 2 Complexity Institute, Nanyang Technological University, Singapore, Singapore; Instituto Nacional de Medicina Genomica, MEXICO

## Abstract

There is growing interest in the use of critical slowing down and critical fluctuations as early warning signals for critical transitions in different complex systems. However, while some studies found them effective, others found the opposite. In this paper, we investigated why this might be so, by testing three commonly used indicators: *lag-1 autocorrelation*, *variance*, and *low-frequency power spectrum* at anticipating critical transitions in the very-high-frequency time series data of the Australian Dollar-Japanese Yen and Swiss Franc-Japanese Yen exchange rates. Besides testing rising trends in these indicators at a strict level of confidence using the Kendall-tau test, we also required statistically significant early warning signals to be concurrent in the three indicators, which must rise to appreciable values. We then found for our data set the optimum parameters for discovering critical transitions, and showed that the set of critical transitions found is generally insensitive to variations in the parameters. Suspecting that negative results in the literature are the results of low data frequencies, we created time series with time intervals over three orders of magnitude from the raw data, and tested them for early warning signals. Early warning signals can be reliably found only if the time interval of the data is shorter than the time scale of critical transitions in our complex system of interest. Finally, we compared the set of time windows with statistically significant early warning signals with the set of time windows followed by large movements, to conclude that the early warning signals indeed provide reliable information on impending critical transitions. This reliability becomes more compelling statistically the more events we test.

## Introduction

Since Scheffer *et al*. published their 2009 [1] and 2012 [2] reviews on *early warning signals* (EWSs) preceding regime shifts, there has been an explosion in the number of papers on this topic for various complex systems. In **Table 1**, we summarized EWS papers published between 2014 and 2017, showing the types of complex systems they were dealing with, and whether they could observe the various EWSs. While most of these papers successfully detected significant EWSs prior to extreme dynamics in their complex systems, a few reported negative results for one or more EWSs [3, 4].We found these negative results intriguing.

**Table 1:** A survey of the EWS literatures between 2014 and 2017, showing the titles and first authors of the papers, corresponding reference numbers, their publication dates, whether the data used was real or simulated, the types of complex systems, the common EWIs, and customized EWIs (if any).

• Title • (First author)	Ref Num	Date	Data	Type of system	AR	V/S	PS	SK	KT	FK	Others or remarks
• Vegetation recovery in tidal marshes reveals critical slowing down under increased inundation • (J. van Belzen)	[5]	Jun 2017	Real	Ecology							Recovery rate +, spatial variance and autocorrelation +/−
• Direct observation of increasing recovery length before collapse of a marine benthic ecosystem • (L. Rindi)	[6]	May 2017	Real	Ecology							Recovery length (spatial indicator) +
• Critical slowing down as an early warning of transitions in episodes of bipolar disorder: A simulation study based on a computational model of circadian activity rhythms • (A. Bayani)	[7]	Jan 2017	Simulated	Biology		+		+			
• Alternative stable states and spatial indicators of critical slowing down along a spatial gradient in a savanna ecosystem • (S. Eby)	[8]	Dec 2016	Real	Ecology	+	+	+	+			Indicators: spatial instead of temporal
• Early warning signals of regime shifts in coupled human-environment systems • (C. Bauch)	[9]	Nov 2016	Simulated	Ecology		+					
• Evaluating early-warning indicators of critical transitions in natural aquatic ecosystems • (A. S. Gsell)	[3]	Nov 2016	Real	Ecology	−	−	−	−			Low reliability and agreement among indicators
• Early warning signals for critical transitions in a thermoacoustic system • (E. A. Gopalakrishnan)	[10]	Oct 2016	Both	Physical	−	+					Conditional heteroskedasticity +
• Rate of forcing and the forecastability of critical transitions • (C. F. Clements)	[11]	Oct 2016	Simulated	Ecology	+	+					Density ratio +, and return rate +
• Early warning signals, nonlinearity, and signs of hysteresis in real ecosystems • (M. A. Litzow)	[12]	Oct 2016	Real	Ecology							Spatial AR +, spatial variability +/−, temporal AR +/−, and temporal variability −
• Early warning signals detect critical impacts of experimental warming • (K. S. Mccann)	[13]	Sep 2016	Real	Ecology	+	+					Recovery rate +
• The Regime Shift Associated with the 2004–2008 US Housing Market Bubble • (J. Tan)	[14]	Sep 2016	Real	Housing	+	+	+	+			
• Early-warning indicators for rate-induced tipping • (P. Ritchie)	[15]	Sep 2016	Simulated	Mathematical Model	+	+					
• Nonlinear manifold learning for early warnings in financial markets • (Y. Huang)	[16]	Aug 2016	Real	Financial							Information metric-based manifold learning (IMML) +
• Detecting early signs of the 2007–2008 crisis in the world trade • (F. Saracco)	[17]	Jul 2016	Real	Economics							Bipartite WTW topology change +
• Early warning of critical transitions in biodiversity from compositional disorder • (C. P. Doncaster)	[18]	Jul 2016	Both	Ecology							Correlation between compositional disorder and biodiversity +
• Percolation-based precursors of transitions in spatially extended systems • (V. Rodriguez-Mendez)	[19]	Jul 2016	Both	Ecology/ General							Indicators for percolation transitions of spatial correlation network +
• Resilience changes in watershed systems: A new perspective to quantify long-term hydrological shifts under perturbations • (M. Qi)	[20]	May 2016	Real	Hydrology							Proposed resiliience indicator, CSD +
• Dynamic bifurcations on financial markets • (M. Kozłowska)	[21]	Mar 2016	Real	Financial	+	+				+	
• Anticipating abrupt shifts in temporal evolution of probability of eruption • (J. Rohmer)	[22]	Feb 2016	Simulated	Geology	−	+		+	+		Density ratio +
• Are critical slowing down indicators useful to detect financial crises? • (H. Gatfaoui)	[23]	Feb 2016	Real	Financial	−	+		+			
• Network based early warning indicators of vegetation changes in a land–atmosphere model • (Z. Yin)	[24]	Feb 2016	Simulated	Ecology	+						Moran’s coefficient +, and interaction network based indicators +
• Lack of Critical Slowing Down Suggests that Financial Meltdowns Are Not Critical Transitions, yet Rising Variability Could Signal Systemic Risk • (V. Guttal)	[25]	Jan 2016	Real	Financial	−	+	−				
• Predictability of critical transitions • (X. Zhang)	[26]	Nov 2015	Simulated	Mathematical Model	+	+					
• Early warnings and missed alarms for abrupt monsoon transitions • (Z. A. Thomas)	[4]	Nov 2015	Real	Climate	−	−					
• Critical Slowing Down as an Early Warning Signal for Financial Crisis? • (C. Diks)	[27]	Sep 2015	Real	Financial	+	+					
• Early warning signals for critical transitions in power systems • (H. Ren)	[28]	Mar 2015	Simulated	Power system	+	+		+		+	
• Critical Slowing Down Governs the Transition to Neuron Spiking • (C. Meisel)	[29]	Feb 2015	Real	Biology	+	+					Recovery rate +
• Early warning signals of Atlantic Meridional Overturning Circulation collapse in a fully coupled climate model • (C. A. Boulton)	[30]	Dec 2014	Real	Climate	+	+					
• Evidencing a regime shift in the North Sea using early-warning signals as indicators of critical transitions • (N. Wouters)	[31]	Oct 2014	Real	Ecology	+	+					
• Critical slowing down as early warning for the onset of collapse in mutualistic communities • (V. Dakos)	[32]	Oct 2014	Simulated	Ecology	+	+					
• Critical slowing down associated with regime shifts in the US housing market • (J. P. L. Tan)	[33]	Feb 2014	Real	Housing	+	+					
• Early warning signals of abrupt temperature change in different regions of China over the past 50 years • (J.-L. Tong)	[34]	Feb 2014	Real	Climate	+						

In this table, a ‘+’ indicates that the EWS was found to be sufficiently significant, a ‘−’ indicates that the EWS was found to be insignificant, and a ‘+/−‘ indicates that the EWS was found to be significant only under certain conditions. In this table, the common EWIs compared are lag-1 autocorrelation (AR), variance/standard deviation (V/S), low-frequency power spectrum (PS), skewness (SK), kurtosis (KT), and flickering (FK). Other EWIs, like recovery rate, spatial variance, spatial correlation, conditional heteroskedasticity for example, are recorded under ‘others or remarks’.

In principle, a complex system approaching a generic regime shift should exhibit EWSs in most, if not all *early warning indicators* (EWIs). However, we also understand that some of these EWIs (for example, the lag-1 autocorrelation) might sit on top of non-critical backgrounds, while others (for example, the skewness) are statistically difficult to measure. In addition, for some EWSs, the tests of statistical significance (for example, the Kendall-tau test) that are used might be too strict. Therefore, as we strive to avoid false positives (statistically significant EWSs but no regime shift), it is also important for us not to throw out the baby with the bath water, by introducing too many false negatives (regime shift with statistically insignificant EWSs). To understand how such a compromise can be struck, we turned our focus on the foreign exchange (FOREX) market, which is the most fluid market in the financial world. We chose to work with FOREX data because (1) the FOREX market is a *bona fide* complex system, with (2) very frequent booms and crashes, and for which (3) very high-frequency data is available. The large number of critical transitions (booms and crashes) means that we can test the performances of the EWIs over many events (instead of only over one large event in many slowly-evolving complex systems). The high data frequency in the raw data means that we can systematically test the performances of the EWSs for the same set of events using different test data frequencies, by omitting more and more raw data points, to simulate lower-frequency data collected for other complex systems.

To better understand the conditions under which we can reliably obtain EWSs for impending critical transitions, we use the Kendall-tau test to examine the significance of rising trends in the three most common EWIs, namely the *lag-1 autocorrelation* (AC(1)), the *variance* (Var), and the *low-frequency power spectrum* (LFPS) while systematically fine tuning three time scale parameters, one de-trending parameter, and one LFPS parameter. We compared the performances of EWSs with different parameters, and obtained optimal combinations of parameters for accuracy as well as timeliness of EWSs. We also experimented with poor choices of parameters and found EWSs either lose reliability or simply disappear. In addition to working with the assumption that EWSs that are concurrent in three EWIs are more reliable than EWSs that do not simultaneously appear in all three EWIs, we quantitatively analyzed the reliability of EWSs, and found that they provide useful information for anticipating incoming critical transitions.

The organization of this paper is as follows. In the **Data and Methods** section, we will describe the FOREX data we used in our studies, and the pre-processing that we have done. We also describe how the three EWIs can be computed, and how we test for statistically significant rising trends in these EWIs. Beyond the rising trends, we also explain why we insist on concurrence between the EWSs, and why the endpoints of the rising EWIs must be large for the EWSs to be predictors of large movements in the FOREX market. We end this section by describing a systematic sensitivity analysis to test how strongly the EWSs depend on our choice of parameters. Following these, in the **Results and Discussion** section, we describe the statistically significant EWSs found in our FOREX data, and the optimal parameter choices that emerged from our sensitivity analysis. We then demonstrate how a poor choice of data frequency can lead to false negatives, before going on to test the reliability of the EWSs, by checking how often a large FOREX market movement follows a statistically significant EWS.

## Data and methods

### Data

We downloaded exchange rates between two pairs of currencies on the foreign exchange (FOREX) market (see **Table 2**) from the Thomson-Reuters Tick History Database (https://tickhistory.thomsonreuters.com/TickHistory/login.jsp). The currencies studied are the Australian Dollar (AUD), the Japanese Yen (JPY), and the Swiss Franc (CHF), while the period studied (1995 to 2010) consists of years within the Global Financial Crisis (2007 to 2009).

**Table 2:** The two foreign exchange pairs: AUD-JPY and CHF-JPY, and the periods their time series data were available over.

Pair	Start Date	End Date	Number of Ticks
AUD-JPY (early)	02 Jan 1995	31 Dec 2004	5,019,035
AUD-JPY (late)	02 Jan 2005	11 Jan 2010	28,477,160
CHF-JPY	11 Jul 2008	31 Dec 2009	4,932,694

Here, a tick is a single transaction.

### Pre-processing

We first imported the raw data from the Tick History comma-separated (CSV) files into Matlab data structures. We then read through the ticks, and extracted exchange rates at fixed time intervals (*T*_0_) (See Text A in S1 Protocol for Matlab script) that we can specify to obtain our time series data.

From the theory of critical transitions [1, 2], we know that as we approach a tipping point, not only will we observe long-term trends in the slow variables, we will also detect a slowing down in the fluctuations of fast variables. When both effects are present, it is difficult to reliably interpret the EWIs. Therefore, it is important to first remove the long-term trends from the time series data. The simplest way to do de-trending is to use a rolling window. However, the local trends obtained this way do not change smoothly from one rolling window to the next. Therefore, we used a Gaussian kernel to smooth the data [4, 23, 27] (See Text B in S1 Protocol for Matlab script). It is also possible for us to use the LOESS method of non-parametric local regression [14], or more sophisticated methods such as the de-trending algorithms used in the detrended fluctuation analysis (DFA) [35], and the empirical mode decomposition [36]. A systematic comparison of the performance of different de-trending methods is outside the scope of this paper.

**Fig 1:**
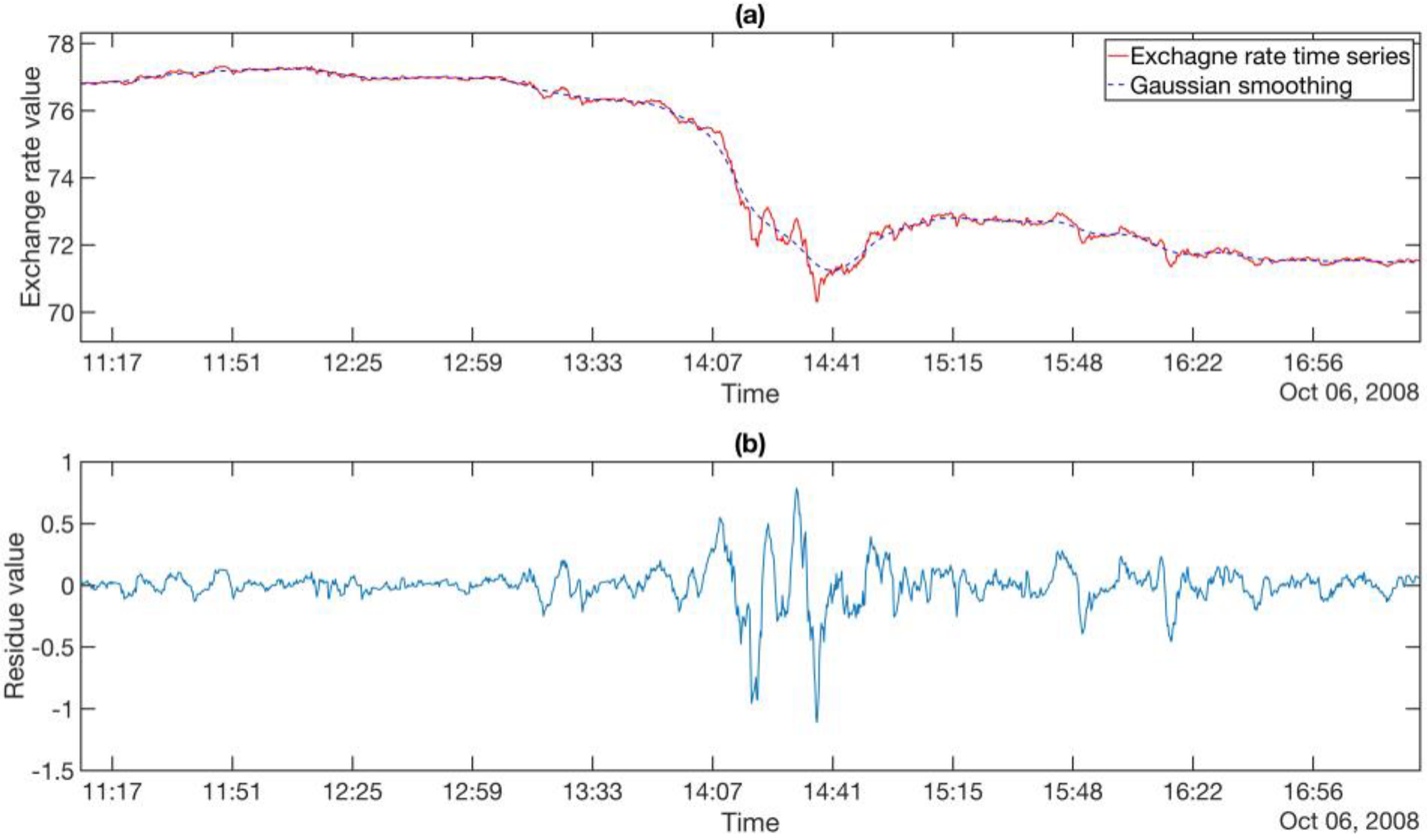
(a) A part of the *T*_0_ = 15 s AUDJPY exchange rate (red) on 6^th^ Oct, 2008, and the Gaussian smoothed time series (blue) that tracks it very closely. (b) The residue time series, obtained by subtracting the Gaussian smoothed time series from the exchange rate. In these plots, the bandwidth of the Gaussian kernel used is 100*T*_0_.

In **Fig 1(A)**, we show the *T*_0_ = 15 s time series for AUDJPY between 11:15 AM and 17:00 PM on Oct 6, 2008 as a red solid curve, and the trend obtained after smoothing with a Gaussian kernel with bandwidth *σ* = 100*T*_0_ as a blue dashed curve. We then show in **Fig 1(B)** the blue residue time series obtained by subtracting the Gaussian-smoothed time series from the exchange rate. Our EWS analysis in the rest of the paper will be based on the residue time series.

### Early Warning Indicators (EWI)

After removing the long-term trends, we tested the residue time series (see **Fig 1(B)**) for critical slowing down. This was done by calculating three EWIs: (1) the *lag-1 autocorrelation* (AC(1))

$$AC(1)=\frac{1}{N-1}\sum_{n=0}^{N-2}\left(x_{n}-\bar{x}\right)(x_{n+1}-\bar{x}),$$

where $\bar{x}=\frac{1}{N}\sum_{i=1}^{N}x_{i}$ is the mean of the sequence (*x*_*n*_), and *N* is the number of total elements in the sequence; (2) the *variance* (Var)

$$Var=\frac{1}{N-1}∙\sum_{i=0}^{N-1}{\left(x_{i}-\bar{x}\right)}^{2}$$

of the sequence (*x*_*n*_); and (3) the *low-frequency power spectrum* (LFPS). Given a sequence (*x*_*n*_), we define its discrete Fourier transformation as

$$X_{k}≝\sum_{n=0}^{N-1}{x_{n}∙e}^{-2\pi ikn/N},$$

where *k* is an integer from 0 to *N* − 1. The power spectrum *P*_*k*_ = |*X*_*k*_|^2^, *k* = 0,…,*N* − 1, is then normalized so that its sum is 1. Finally, the LFPS is then calculated to be the power residing in the first 6% elements of the sequence *P*_*k*_. The LFPS of a sequence (*x*_*n*_) measures the weightage of the low-frequency part in the power spectrum.

### Testing for significant EWSs

#### Increasing trends

When we slide a rolling window of length *R*_*win*_ (corresponding to a window duration of *T*_1_ = *R*_*win*_ ∙ *T*_0_) over the entire residue time series with rolling step *R*_*step*_ (set to be one third of *R*_*win*_), we create the time series of indicators. For all three indicators, an EWS corresponds to an increasing trend in the indicator values. Therefore, we test EWSs for statistical significance within rolling windows of indicators of length *R*_*ind*_ (corresponding to a window duration of *T*_2_ = (*R*_*ind*_ ∙ (*R*_*step*_ −1) + *R*_*win*_) ∙ *T*_0_) with rolling step 1 along the time series of the three indicators respectively.

Within each rolling window of length *N* = *R*_*ind*_, we calculate the Kendall-tau values, also known as the Kendall rank correlation coefficient [37],

$$\tau =\frac{N_{concordant\;pairs}-N_{disconcordant\;pairs}}{N(N-1)/2},$$

where *N*_*concordant pairs*_ is the total number of concordant pairs and *N*_*disconcordant pairs*_ is the number of disconcordant pairs. Suppose *t*_1_ < *t*_2_, the pair of $\left(x_{t_{1}},t_{1}\right)$ and $\left(x_{t_{2}},t_{2}\right)$ is said to be concordant if $x_{t_{1}}<x_{t_{2}}$ and discordant if $x_{t_{1}}>x_{t_{2}}$. Note that if $x_{t_{1}}=x_{t_{2}}$, the pair is neither concordant nor discordant.

To see how this works, let us consider the ordered series: (2, 4, 3, 8). Except for 4 coming before 3, the rest of the series has an increasing trend. To see this using the Kendall-tau coefficient, we note that according to the definition, there are 5 concordant pairs: (2, 4), (2,3), (2, 8), (4, 8), (3, 8), and 1 disconcordant pair: (4, 3). In total there are $\frac{4(4-1)}{2}=6$ pairs. In this example, the Kendall-tau coefficient is $\frac{\left(5-1\right)}{6}=\frac{2}{3}$, which is fairly large. In general, a time series with a strong increasing trend will have a high Kendal-tau coefficient.

To determine the statistical significance of the Kendall-tau value of a given rolling window of indicators, which has *R*_*ind*_ indicators corresponding to *R*_*ind*_ ∙ (*R*_*step*_ −1) + *R*_*win*_ data points in the residue time series, we first reshuffle the residue time series of *R*_*ind*_ ∙ (*R*_*step*_ −1) + *R*_*win*_ data points, to create a null model residue time series that has the same mean and variance as the subject time series, but whose time ordering is completely destroyed. Here, let us point out that normally, to test the Kendall-tau of the **indicator** time series for statistical significance we reshuffle the **indicator** time series. By reshuffling the **residue** time series instead, we are making the significance tests stricter. We repeat this procedure 1000 times to create a histogram of 1000 Kendall-tau values for the null model. The *p* value of the subject Kendall-tau is then the percentage of null-model Kendall-tau values that are greater than the subject Kendall-tau value. For the purpose of this paper, if *p* ≤ 0.05, we regard the EWS in this time interval as significant.

#### Concurrence

A period with one statistically significant EWI points to an impending critical transition. However, the other EWIs may not be statistically significant over the same period, or they may be statistically significant over slightly different periods. Since it is possible for a statistically significant EWS to be a false positive, we can reduce the false-positive rate by requiring all three EWIs to be statistically significant over the same overlapping period. With this concurrent set of EWIs, the probability of the overlapping period being a statistical false positive should be significantly reduced.

#### Endpoint

**Fig 2:**
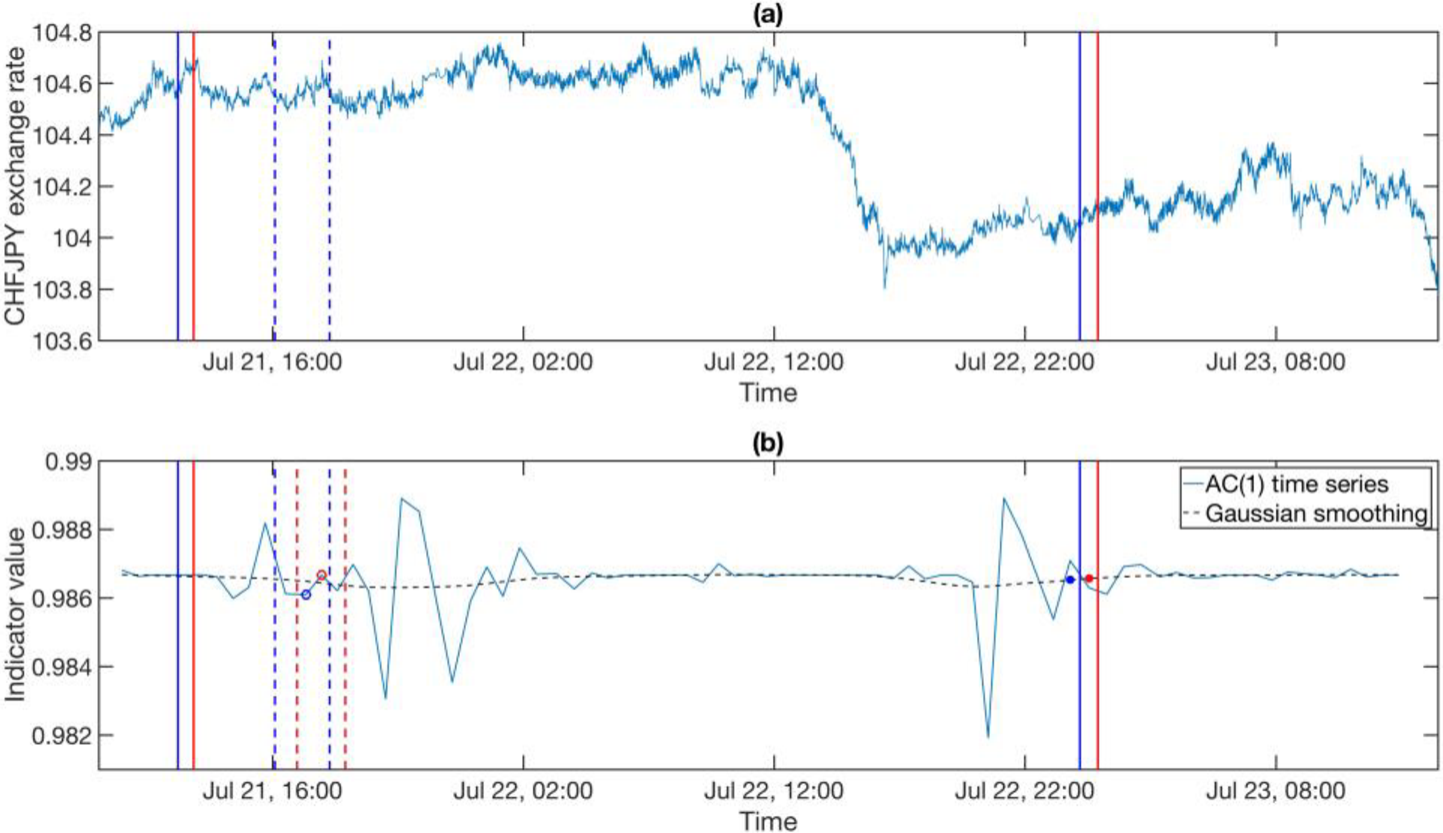
(a) The exchange rate time series and (b) the indicator time series. The pair of blue dashed vertical lines represents the time window over the exchange rate time series in (a) was used to compute one indicator value (the open blue circle in (b)). We slide this window along to obtain the pair of red dashed vertical lines, within which we obtain a second indicator value (the open red open circle in (b)). Repeating this we obtained the indicator time series in (b), which is then Gaussian smoothed (blue dashed curve). For the *T*_2_ rolling window indicated by the pair of blue solid vertical lines, its endpoint is the last value on the Gaussian-smoothed indicator time series (the solid blue dot in (b)). The endpoint value of the next *T*_2_ rolling window (the pair of red solid vertical lines) is shown in (b) as the solid red dot on the Gaussian-smoothed curve.

Sometimes we encounter situations where the rising trends of the indicators are statistically significant but the indicators values remain small at the end of the *T*_2_ time windows. We show in **Fig 2** the magnitude of last indicator value in a *T*_2_ time window, and call it the *endpoint* of the indicator. If the endpoint is small, we do not expect to find a critical transition shortly after the EWS even if the rising trend is significant. We expect a critical transition only if the rising trend is statistically significant *and* the endpoint is large.

**Fig 3:**
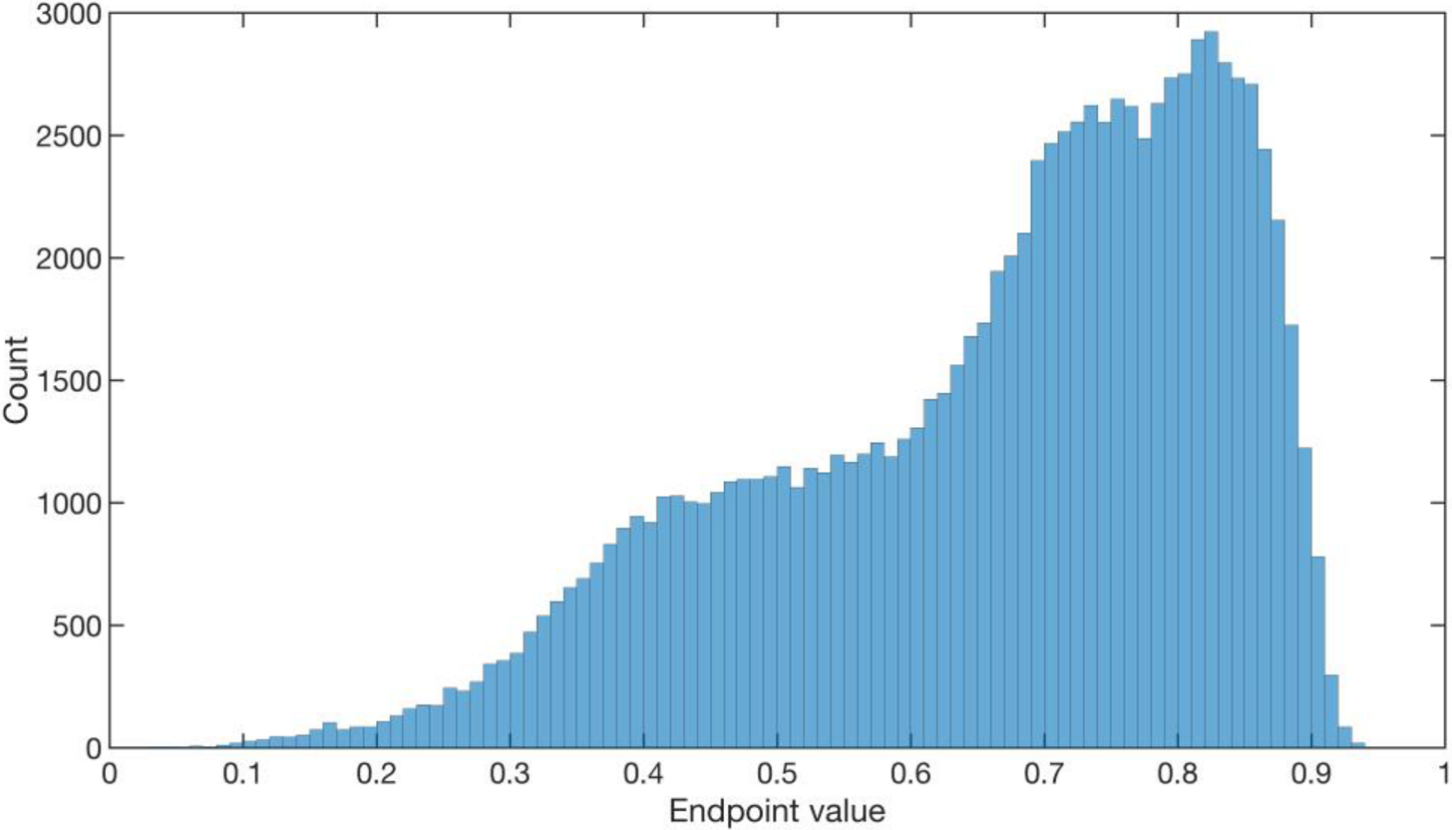
The histogram of endpoints of *T*_2_ rolling windows over the entire AUD-JPY time series from 2005 to 2010.

To decide whether the endpoint is large or small, we build the histogram shown in **Fig 3** of the endpoints of *T*_2_ rolling windows over the entire time series. The ‘historical *p* value’ of the endpoint of an EWS candidate is the percentage of endpoints in the histogram that are larger than it. Only endpoints within lowest historical *p* values are considered as EWS candidates. A more careful reliability analysis will be presented at the end of the **Results and Discussion** section.

### Choice of parameters and sensitivity analyses

#### Choice of parameters

In this study, the parameters we have freedom to adjust are summarized in **Table 3**.

**Table 3:** Parameters and their test ranges to determine the optimal combination for EWSs, as well as to test the effects of data frequency.

Parameters	Meaning	Range of values for finding optimal combination	Range of values for testing the effect of data frequency
*T*_0_	Time interval between residue data points	15–60 s	15 s to 6 hrs
*R*_*win*_	The number of residue data points in a rolling window used to compute one indicator value	60–249	10–14400
*R*_*step*_	The rolling step of the rolling window of residue	Fixed to be *R*_*win*_/3	Fixed to be *R*_*win*_/3
*R*_*ind*_	The number of indicator data points in a rolling window used to test statistical significance	92–96	10 (fixed)
*R*_1_	The rolling step of the rolling window for indicator time series	1	1
*σ*	The bandwidth of the Gaussian kernel for smoothing the residue time series	38–180	20–150
∑	The bandwidth of the Gaussian kernel for smoothing the indicator time series	Fixed to be *R*_*ind*_	Fixed to be *R*_*ind*_
*P*	The percentile of power spectrum defining the low-frequency power	3%–38%	10% (fixed)

#### Sensitivity analyses

We performed two sets of sensitivity analyses in this paper. In the first, we determined the optimal combination of parameters to detect EWSs of large movements in the FOREX market.

To do this, we must identify the events we sought to forecast. In order to quantitatively pick out sudden shifts in the exchange rate, we consider a time period *Y* starting from the end of the *T*_2_ rolling window, to half a day afterwards, and define the *maximum spread* to be

$$y_{ms}=\frac{\max(E_{0}-E_{\min},E_{\max}-E_{0})}{E_{0}}.$$

Here, *E*_0_ is the exchange rate at the beginning of time period *Y*, *E*_min_ is the minimum exchange rate within *Y*, and *E*_max_ is the maximum exchange rate within *Y*. Basically, *y*_*ms*_ measures the most extreme exchange rate variation within *Y*, relative to its starting value, allowing variations in either directions. This can be *E*_0_ − *E*_min_, if it is larger than *E*_max_ – *E*_0_, or *E*_max_ – *E*_0_ vice versa. Higher values of *y*_*ms*_ correspond to more extreme exchange rate variations within *Y*.

**Fig 4:**
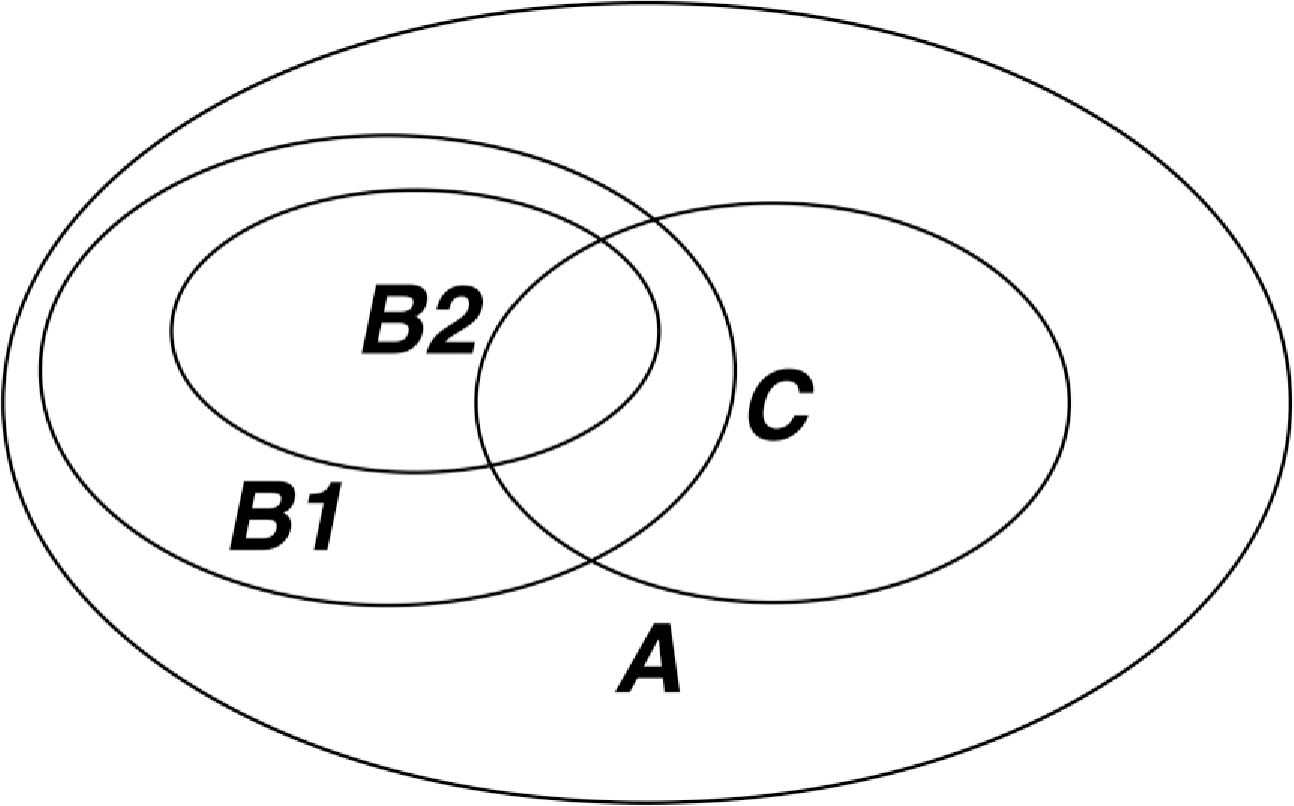
Venn diagram illustration for maximum spreads, in terms of percentile and statistical significance. In this illustration, *A* represents the set of maximum spreads {*y*_*ms*_} over the entire time series using a given combination of parameters. *B1* and *B2* represent the elements in *A* with *y*_*ms*_ values above 90^th^ percentile and 95^th^ percentile. *C* represents the set of the maximum spreads corresponding to statistically significant EWSs.

**Fig 5:**
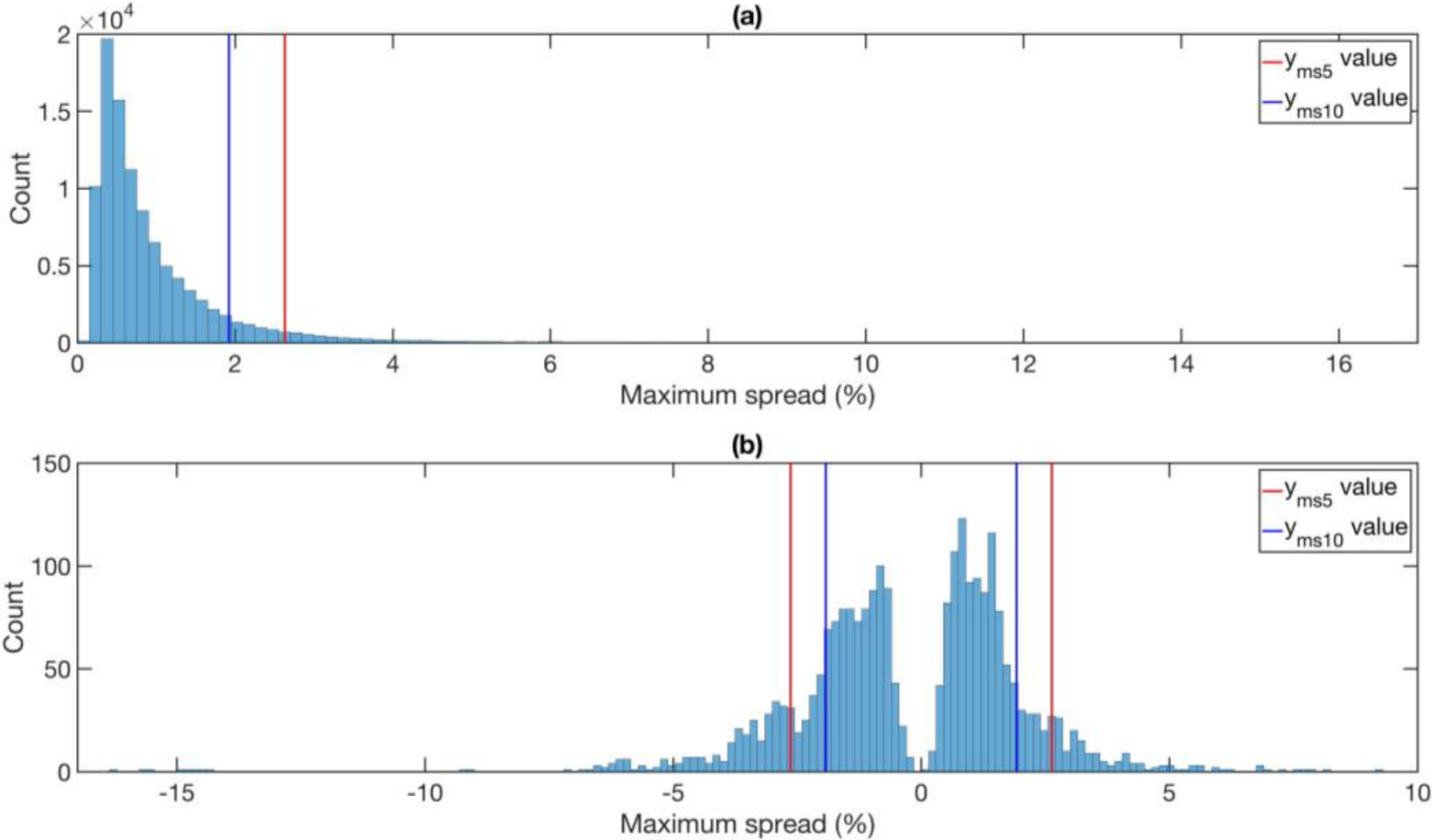
**(a) The histogram of maximum spreads *y***_***ms***_ **in Set A. (b) The histogram of maximums spreads *y***_***ms***_ **in Set A with signs (positive for boom and negative for bust).** The blue and red solid vertical lines indicate the 90^th^ and 95^th^ percentiles *y*_*ms*10_ and *y*_*ms*5_ respectively. The maximum spread is defined to be positive, but in this histogram, we restore their signs. We can think of a large positive maximum spread as a boom, and a large negative maximum spread as a bust. The blue and red solid vertical lines in (b) correspond to the signed *y*_*ms*10_ and *y*_*ms*5_ values respectively.

To examine the performance of a certain combination of parameters, we first created the sets *A*, *B1*, *B2*, and *C* as shown in **Fig 4**. The corresponding 90^th^ percentile and 95^th^ percentile values of *y*_*ms*_ are noted as *y*_*ms*10_ and *y*_*ms*5_ respectively (shown as the vertical blue line and the vertical red line in **Fig 5(A)**). Based on the intersections *C* ∩ *B*1 = {*y*_*ms*_ ∈ *C*|*y*_*ms*_ > *y*_*ms*10_} and *C* ∩ *B*2 = {*y*_*ms*_ ∈ *C*|*y*_*ms*_ > *y*_*ms*5_} as shown in **Fig 5(B)**, we defined the 5% and 10% discovery rates of Set *C*, *DR*_5_ and *DR*_10_, as

$${DR}_{5}=\frac{card(C\cap B2)}{card(A)},$$

$${DR}_{10}=\frac{card(C\cap B1)}{card(A)},$$

where *card*() stands for cardinality, which is the number of elements in the set. We also defined the 5% and 10% specificities of Set *C*, *SP*_5_ and *SP*_10_, as

$${SP}_{5}=\frac{card(C\cap B2)}{card(C)},$$

$${SP}_{10}=\frac{card(C\cap B1)}{card(C)}.$$

In this analysis, our objective was to choose parameters that maximize discovery rates and specificities.

In our survey of the literature on EWSs, we noticed that most studies confirmed EWSs preceding critical transitions, while other studies could not detect statistically significant EWSs. However, the qualities of data used in these analyses are highly uneven, in the sense that in some studies, very high frequency data was used, whereas in other studies, the data frequency was low. Because we had the good fortune of working with FOREX data at the highest frequency, we could create data samples over many orders of magnitude in data frequency. Therefore, in this second sensitivity analysis, we systematically test the effect of data frequency (determined by *T*_0_) on the discoverability of a subset of very obvious true positives. A true positive is discoverable at a given data frequency if there are statistically significant EWSs preceding the true positive. In this analysis, we fixed the largest window size *T*_2_, but kept the product of *T*_1_ and *T*_0_ constant so that the number of indicator values used for significance testing (*R*_*ind*_) is fixed (at 10), as we increased *T*_0_ from the optimum (15 s or 30 s) to the very large value of 6 hrs.

## Results and discussion

### Results

**Fig 6:**
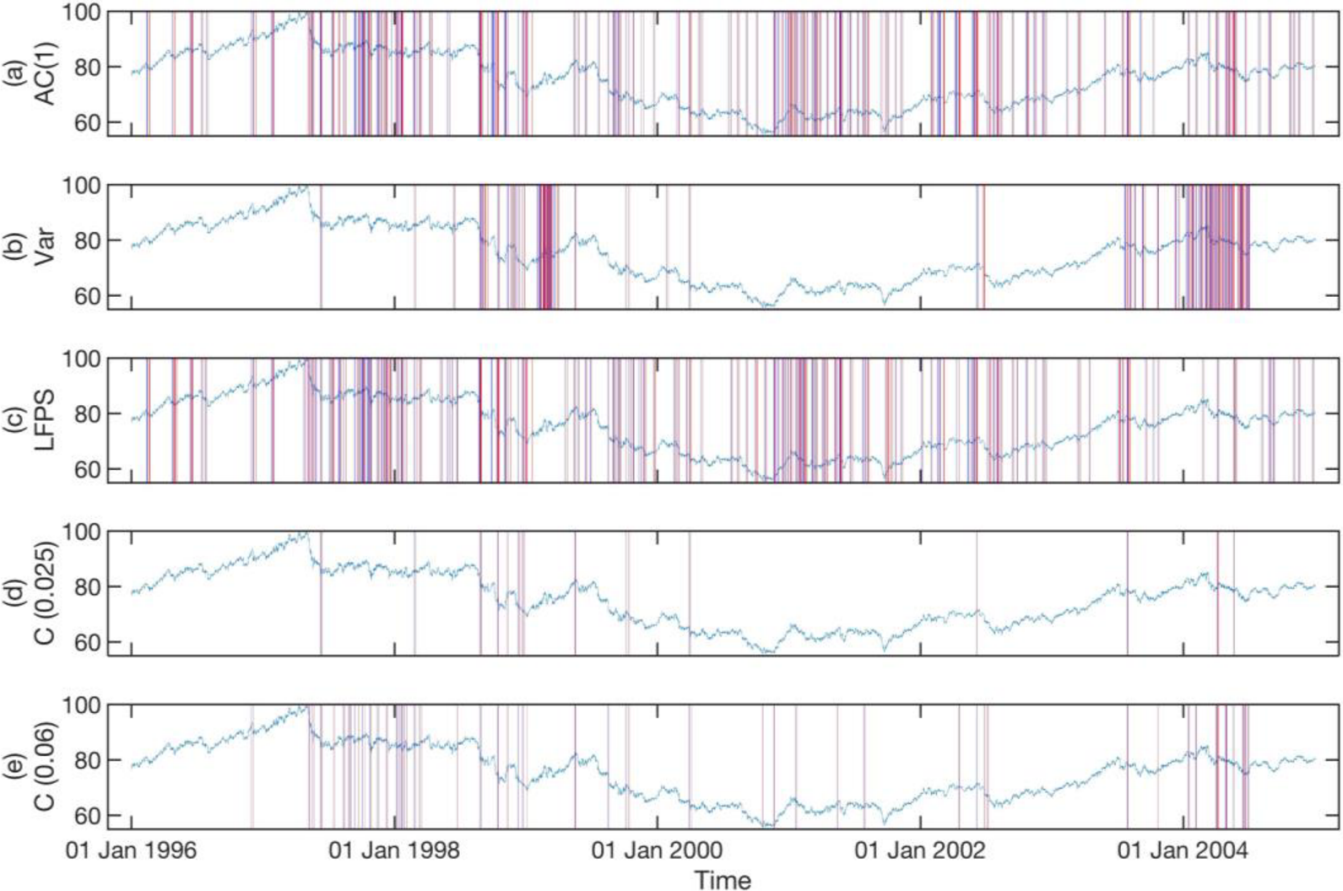
Statistically significant EWSs for the AUD-JPY exchange rate between 1996 and 2004, obtained from the (a) lag-1 autocorrelation, (b) variance, (c) low-frequency power spectrum, (d) concurrent signals with historical p value for endpoint < = 0.025, and (e) concurrent signals with historical p value for endpoint < = 0.06. For subplot (a), (b), and (c), we used a historical *p* value for the endpoint of 0.025. In (d), we show the concurrent EWSs for the same historical *p* value. More statistically significant concurrent EWSs can be included in (e) by increasing the historical *p* value of the endpoint to *p* ≤ 0.06. In this figure, a statistically significant EWS begins at a solid blue vertical line and ends at a solid red vertical line.

**Fig 7:**
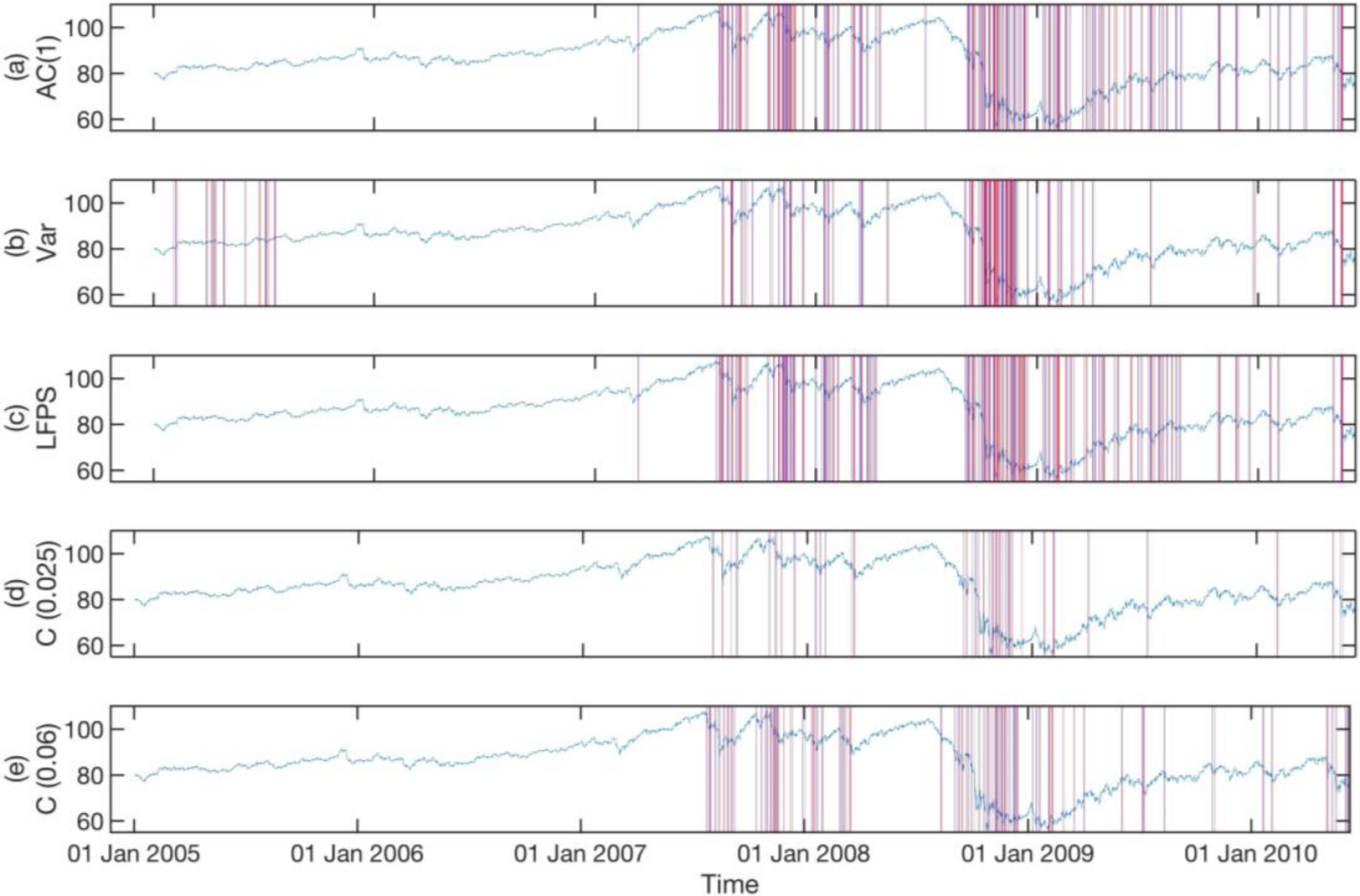
**Statistically significant EWSs for the AUD-JPY exchange rate between 2005 and 2010, obtained from the (a) lag-1 autocorrelation, (b) variance, (c) low-frequency power spectrum, (d) concurrent signals with historical p value for endpoint < = 0.025, and (e) concurrent signals with historical p value for endpoint < = 0.06.** For subplot (a), (b), and (c), we used a historical *p* value for the endpoint of 0.025. In (d), we show the concurrent EWSs for the same historical *p* value. More statistically significant concurrent EWSs can be included in (e) by increasing the historical *p* value of the endpoint to *p* ≤ 0.06. In this figure, a statistically significant EWS begins at a solid blue vertical line and ends at a solid red vertical line.

**Fig 8:**
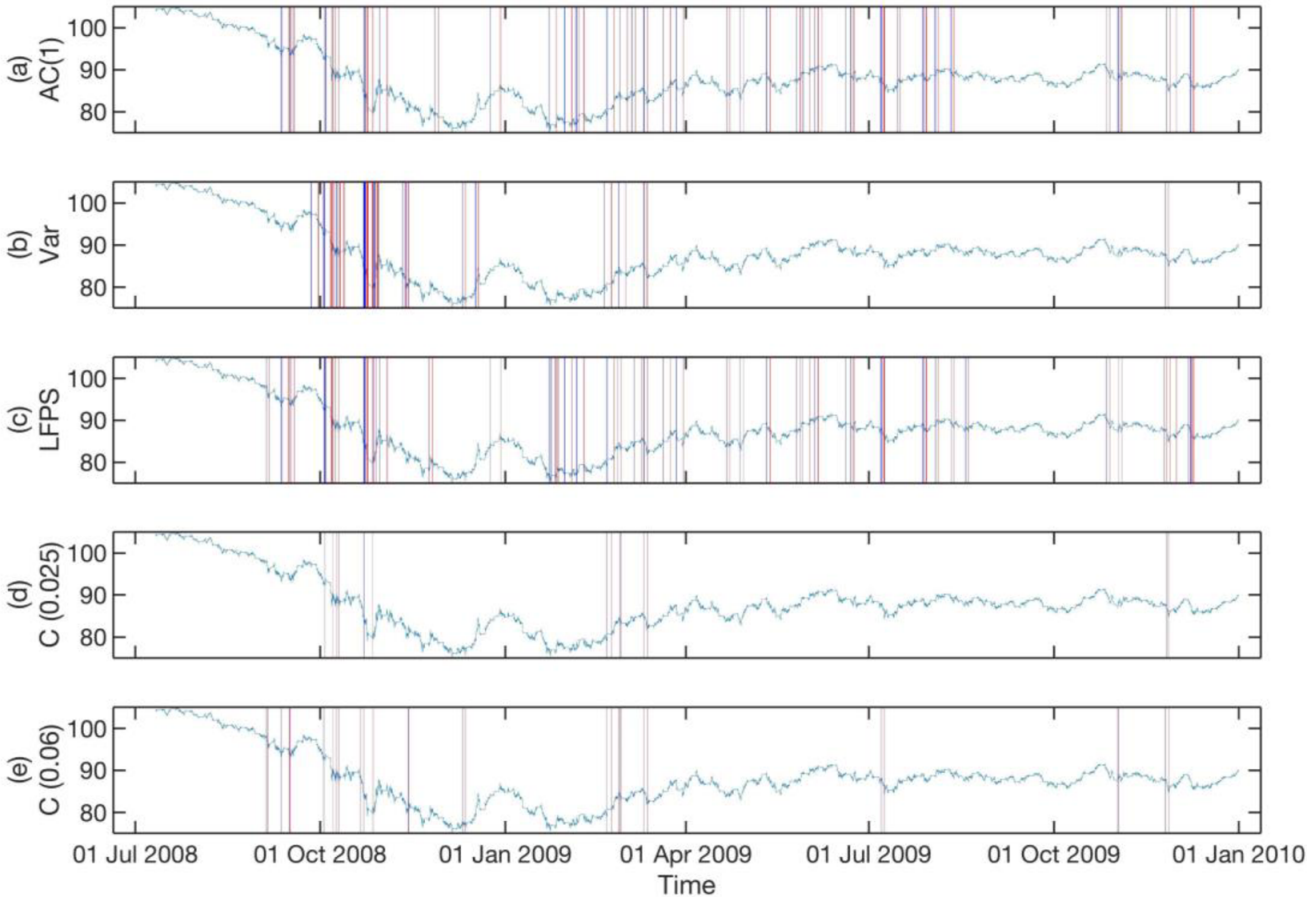
**Statistically significant EWSs for the CHF-JPY exchange rate between 2008 and 2009, obtained from the (a) lag-1 autocorrelation, (b) variance, (c) low-frequency power spectrum, (d) concurrent signals with historical p value for endpoint < = 0.025, and (e) concurrent signals with historical p value for endpoint < = 0.06.** For subplot (a), (b), and (c), we used a historical *p* value for the endpoint of 0.025. In (d), we show the concurrent EWSs for the same historical *p* value. More statistically significant concurrent EWSs can be included in (e) by increasing the historical *p* value of the endpoint to *p* ≤ 0.06. In this figure, a statistically significant EWS begins at a solid blue vertical line and ends at a solid red vertical line.

In **Figs 6–8**, we show the statistically significant EWSs obtained from individual EWIs (See Texts C, D, and E in S1 Protocol for Matlab script) with historical *p* values of their endpoints set to *p* ≤ 0.025 (See Text F in S1 Protocol for Matlab script), compared to concurrent EWSs (See Text G in S1 Protocol for Matlab script) with the same historical *p* value for the three different data sets. In these three figures, we also show the concurrent EWSs for a historical *p* value of their endpoints set to *p* ≤ 0.06, to illustrate how we can include more statistically significant EWSs. The parameters used to detect the EWSs are the optimal combinations for the three data sets. We will explain how these optimal parameter combinations are obtained shortly.

In **Fig 7**, the numbers of statistically significant EWSs predicted by the three indicators are roughly equal. Also, the statistically significant EWSs predicted by Var are mostly at similar times to those predicted by AC(1) and LFPS. However, in **Figs 6** and **8**, even though the numbers of statistically significant EWSs predicted by Var are roughly the same as those predicted by AC(1) and LFPS, those predicted by Var are concentrated in a small number of time periods. We believe this is because the variations of AC(1) and LFPS are within a narrow band of values, whereas the variations of Var can be over many orders of magnitude. Therefore, the condition for a strict historical *p* value for the endpoint of Var restricts the discovery of statistically significant EWSs to only periods with very high variance.

From **Figs 6–8**, we see that the effect of relaxing the historical *p* value for the endpoints is that the additional concurrent EWSs being included are mostly close to those already included at the stricter historical *p* value. This gives us confidence that the EWSs are indeed consistent precursors to actual critical transitions. In fact, the bunching up of EWSs seen in the figures is consistent with the general pattern of flickering critical transitions being preceded by foreshocks and followed by aftershocks. More importantly, the sharpest decline in AUD-JPY exchange rate on 6 Oct 2008 in **Fig 7** is preceded by consistent EWSs in all indicators and therefore shows up strongly in the concurrent EWSs.

### Optimal combination of parameters

The sets of EWSs discovered depend on the parameter combinations that we used. Therefore, we performed the sensitivity analyses, where parameters are sequentially optimized for high discovery rates and specificities, as shown in **Tables A, B, and C in S1 Appendix**. From these tables, we concluded the optimal parameter combinations for AUD-JPY from 1996 to 2004, AUD-JPY from 2005 to 2010, and CHF-JPY from 2008 to 2009 to be (*T*_0_, *σ*, *R*_*win*_, *R*_*ind*_, *P*) = (30, 76, 126, 86, 6), (15, 100, 225, 80, 26), and (30, 48, 150, 84, 24) respectively. (See **Text H in S1 Protocol** for Matlab script)

Most of the time, a 1% change in the parameters around the optimal values in **Tables A, B, and C in S1 Appendix** produces less than 1% change in the discovery rate (DR5) and specificity (SP5) (see **Tables D in S1 Appendix**). The discovery rate and specificity are most sensitive to changes in *R*_*ind*_ and *R*_*win*_, although the percentage changes are still small.

### Effects of increasing time interval

Following this, we turned our attention to the key question in this paper: whether the EWSs can always be detected in lower-frequency data. In this analysis, we focused on increasing the time interval from 15 s to 6 hr (see **Table 4**), checking if the EWSs discovered at optimal *T*_0_ (15 s and 30 s) were also discovered at longer time intervals.

**Table 4:** Parameter combinations with *T*_0_ increasing from the optimal value up to 6 hr, to test whether the EWSs can be discovered at longer time intervals.

*T*_0_	*R*_*win*_	*R*_*ind*_	*σ*
15 sec	14400	10	150
30 sec	7200	10	60
1 min	3600	10	30
2 min	1800	10	24
5 min	720	10	20
10 min	360	10	20
20 min	180	10	20
40 min	90	10	20
1 hr	60	10	20
2 hr	30	10	20
3 hr	20	10	20
4 hr	15	10	20
5 hr	12	10	20
6 hr	10	10	20

**Fig 9:**
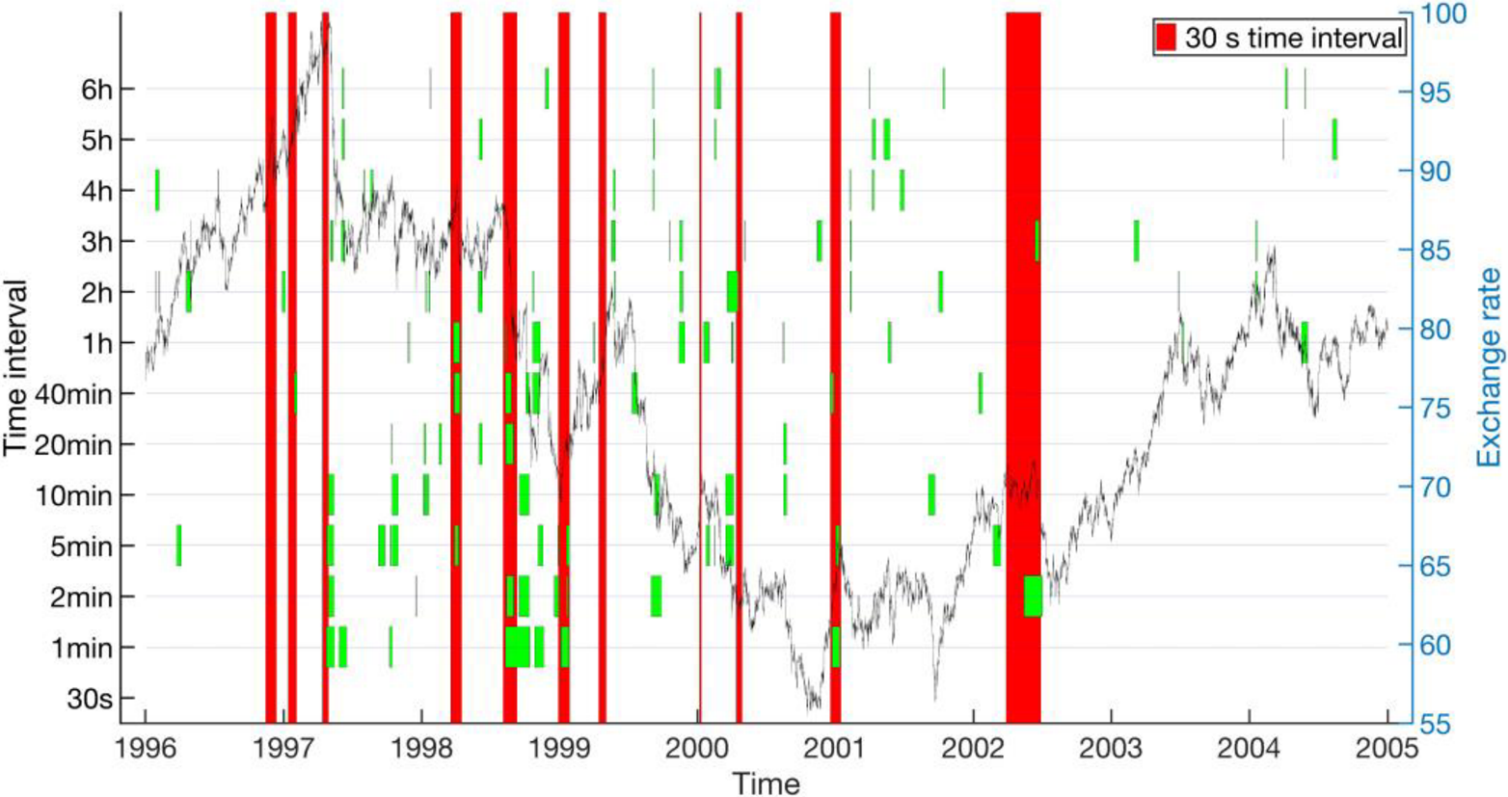
Concurrent EWSs (short green bands) for various time intervals (left axis) compared with the EWSs for 30-s time interval (long red bands) for AUD-JPY from 1996 to 2004. The historical endpoint requirement is *p* ≤ 0.2. The exchange rate is plotted (black curve) is plotted in the background with axis to the right.

**Fig 10:**
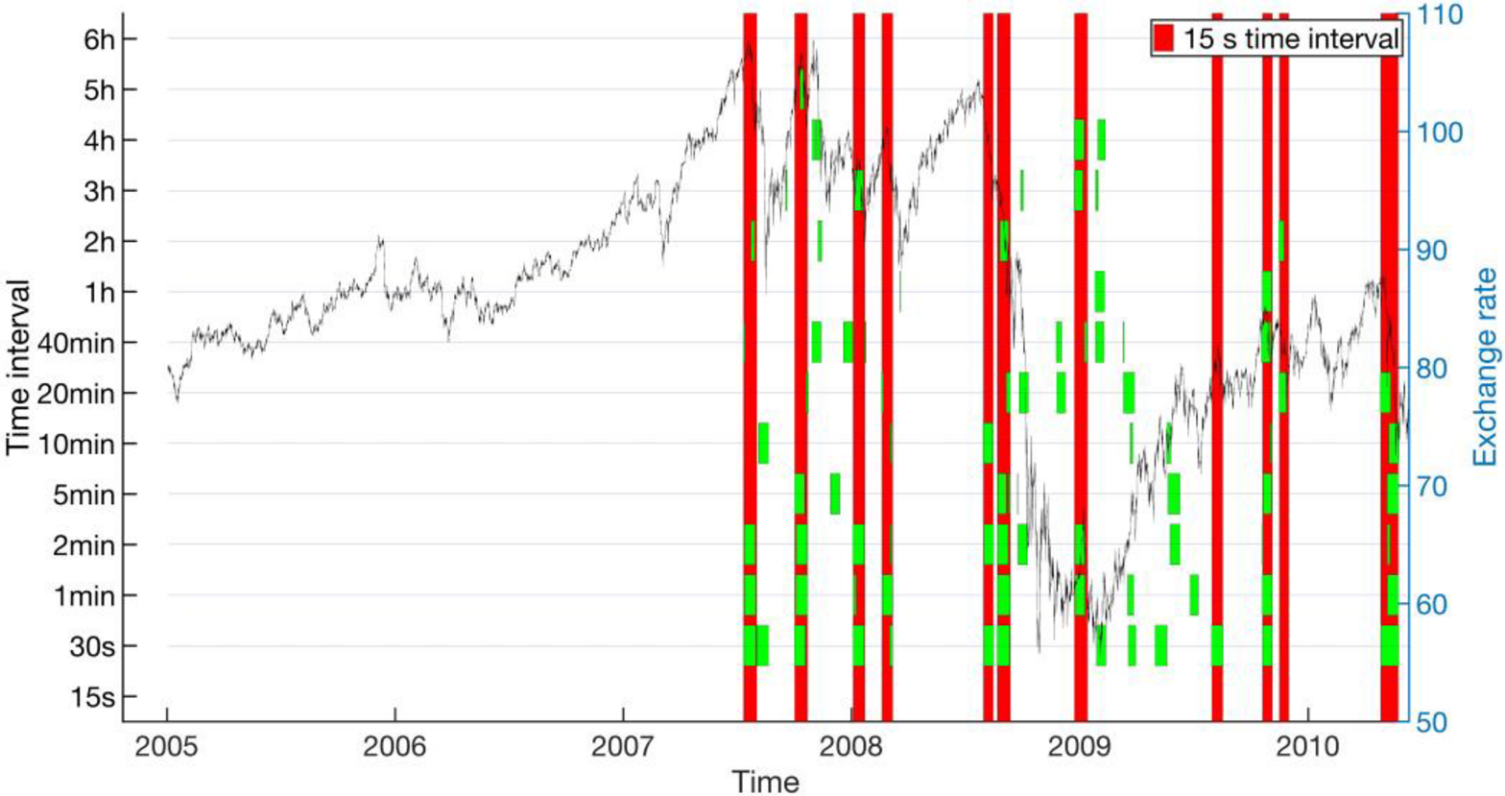
Concurrent EWSs (short green bands) for various time intervals (left axis) compared with the EWSs for 15-s time interval (long red bands) for AUD-JPY from 2005 to 2010. The historical endpoint requirement is *p* ≤ 0.2. The exchange rate is plotted (black curve) is plotted in the background with axis to the right.

**Fig 11:**
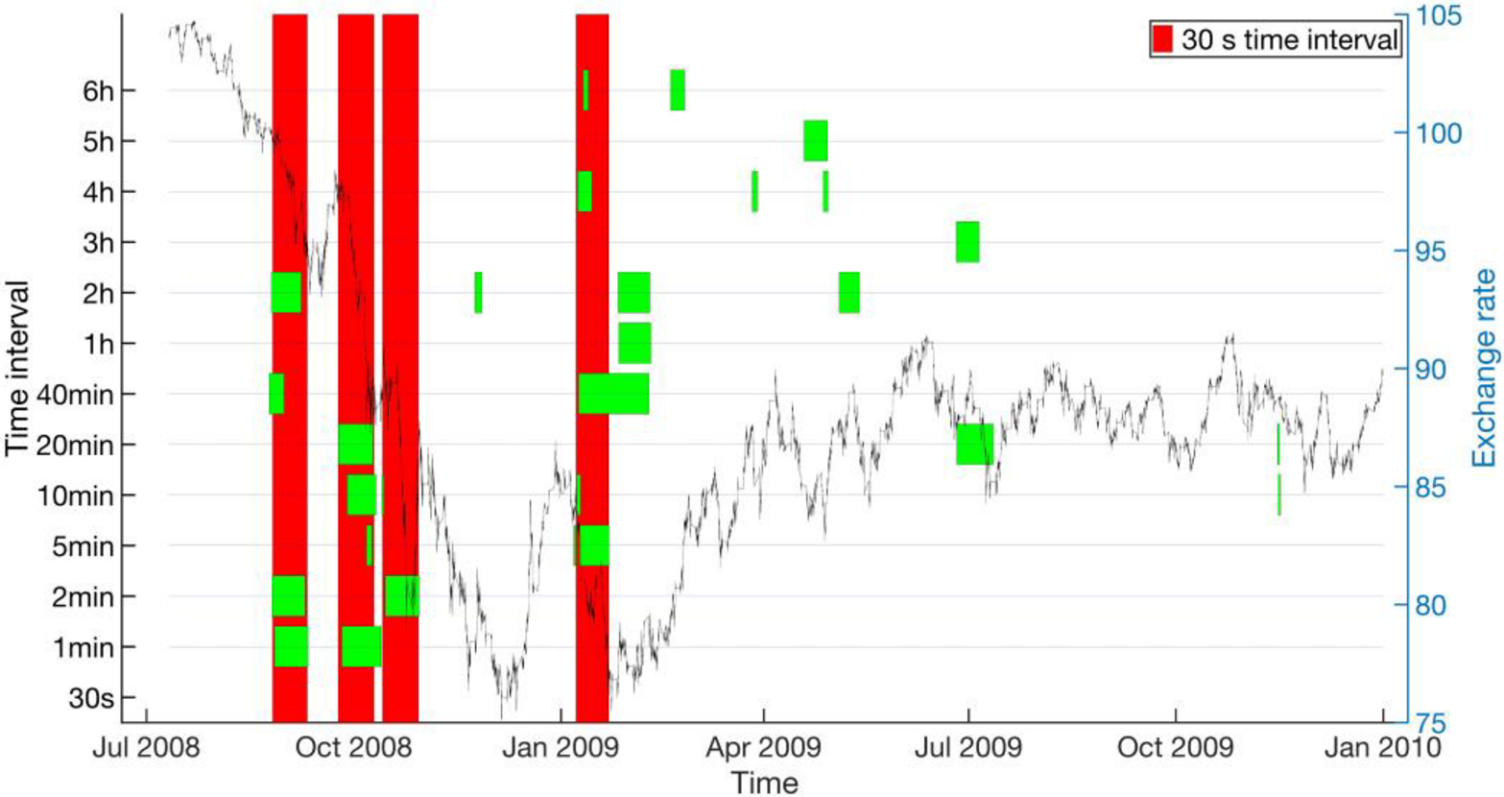
Concurrent EWSs (short green bands) for various time intervals (left axis) compared with the EWSs for 30-s time interval (long red bands) for CHF-JPY from 2009 to 2009. The historical endpoint requirement is *p* ≤ 0.2. The exchange rate is plotted (black curve) is plotted in the background with axis to the right.

As we can see from **Fig 9**, for the AUD-JPY data set from 1996 to 2004, where we used the 30-s EWSs as the ground truth, around half of the critical transitions do not have reliable EWSs at larger *T*_0_’s, whereas for the other half, EWSs are reliable up to around 5 min, above which the EWSs becomes intermittent, disappearing above certain *T*_0_’s. Similarly for **Fig 10**, for the AUD-JPY data set from 2005 to 2010, most of the 15-s EWSs we used as the ground truth can be detected reliably up to 2 min. Beyond *T*_0_ = 10 min, most of the 15-s EWSs do not show up anymore. In the time period when the exchange rate is low, many other EWSs are discovered by the other *T*_0_’s. Finally, from **Fig 11**, for the CHF-JPY data set from 2008 to 2009, the EWSs at larger *T*_0_’s are generally inconsistent with the ground truth at 30 s, especially for *T*_0_’s greater than 2 min.

For the FOREX market, whose dynamical time scale is of the order of 1 to 5 seconds, and where the largest crash is over in a matter of 10 to 15 minutes (see **Fig 1(A)**), it is surprising that we could even have semi-reliable EWSs with data frequencies up to 2 minutes! When we zoom in to **Fig 7** for a closer look, we find that there were up to 3 days of EWSs before the largest crash on 6 Oct 2008. Going through the parameter combinations in **Table 4**, we inferred that these signals were fully captured by the last rolling window, and partially captured by the second last rolling window. This means that out of the ten indicator values that went into the Kendall-tau test, only the last two indicator values contained contributions from the actual EWSs. For time intervals beyond 2 minutes, the 3 days’ worth of EWSs were also only captured by the last two rolling windows. But because fewer data points containing early warning information were sampled during these 3 days, the signal-to-noise ratio becomes smaller. Therefore, we deduced that the deterioration of EWSs for larger time intervals is the result of under-sampling of residue data points within the EWS periods. In other words, low data frequency could significantly compromise the performance of EWSs.

As a caveat, let us note that in this test, we used unusually large 600-hr rolling windows for all time intervals *T*_0_. This was to accommodate the largest 6-hr time interval that we included in the test. Technically, the EWSs presented here are not the most reliable, because they are obtained in a way that is far from ideal, resulting in only a few of them that are sparsely distributed in time. This is unlike the robust consecutively EWSs within proper time periods for the optimal combination of parameters. Moreover, the 600-hr rolling window is much larger than the 3-day period of actual EWS, which therefore must stand out against more noise from the rest of the rolling window. Additionally, to make comparisons, we also had to relax the criteria for historical *p* value to be able to detect a decent number of EWSs. In so doing, even the reliability of EWSs from residue time series at time intervals below 2 minutes is not as high as that of EWSs obtained with the optimal combinations. Nevertheless, the test convincingly shows that large time intervals (beyond 2 minutes) could not produce reliable EWSs. The reliability of EWSs whose time intervals are within 2 minutes were not verified in this section, however we do know that the optimal time interval ranges from 15 seconds to 30 seconds, from the optimal combinations in the previous section.

### Reliability analysis

Finally, to quantify the performance of our EWSs, we examined the conditional probability for a large maximum spread to occur after an EWS, as well as that for a large maximum spread to occur without an EWS. To do so, we examined the maximum spreads (*y*_*ms*_) by the end of every rolling window that was used for computing EWSs (defined by optimal *R*_*win*_ and *R*_*step*_) across the whole time period, and check: (1) whether the maximum spread is within the top 5 percentile, and (2) whether such a large maximum spread is preceded by at least one recent EWS, with *p* < 0.05 for Kendall-tau and the historical *p* < 0.04 for the endpoints. By ‘recent’, we mean that the EWS ended within the last 0.9 days (excluding weekends), even though it may have started much earlier. The maximum spreads *y*_*ms*_ are computed within the time window of 0.1 day starting from the end of every *R*_*win*_ rolling window. We chose to have the time between the end of the EWS and the end of the maximum spread time window to be one day as one day is expected to be a reasonable time to make a decision in this highly liquid FOREX market. To be fair, we used the same 0.1-day time window for large maximum spreads that are not preceded by a recent EWS.

From the pool of all *R*_*win*_ rolling windows, we estimate *P*_1_ and *P*_2_ as

$$P_{1}=P(Large\;y_{ms}|EWS)=\frac{Number\;of\;cases\;with\;large\;y_{ms}\;and\;recent\;EWSs}{Number\;of\;cases\;with\;recent\;EWSs},$$

$$P_{2}=P(Large\;y_{ms}|No\;EWS)=\frac{Number\;of\;cases\;with\;large\;y_{ms}\;and\;no\;recent\;EWSs}{Number\;of\;cases\;with\;no\;recent\;EWSs}.$$

If *P*_1_ = 1, all EWSs should be followed by large (top 5 percentile) maximum spreads. This means that the EWSs provide very *precise* predictions on subsequent exchange rate movements. If *P*_1_ < 1, then some EWSs are not followed by large maximum spreads, so overall the EWSs are less precise. If we act on them to short the exchange rate in question, we may lose the opportunity to make a killing shortly afterwards, but we will not sustain unexpectedly large losses. On the other hand, there can also be large maximum spreads that occur in the absence of EWSs. We can incur large losses if we believe wholeheartedly that no EWSs mean no large maximum spreads afterwards. The proportion of such events, out of the set of cases with no recent EWSs is given by *P*_2_. From all indicators in all data sets, we found that *P*_2_ is at most 0.05. For the EWSs to provide *reliable* predictions, it is necessary to have *P*_1_ > *P*_2_. In fact, the larger the ratio $\frac{P1}{P2}$, the more confident we are at avoiding losses when we act upon the EWSs.

**Fig 12:**
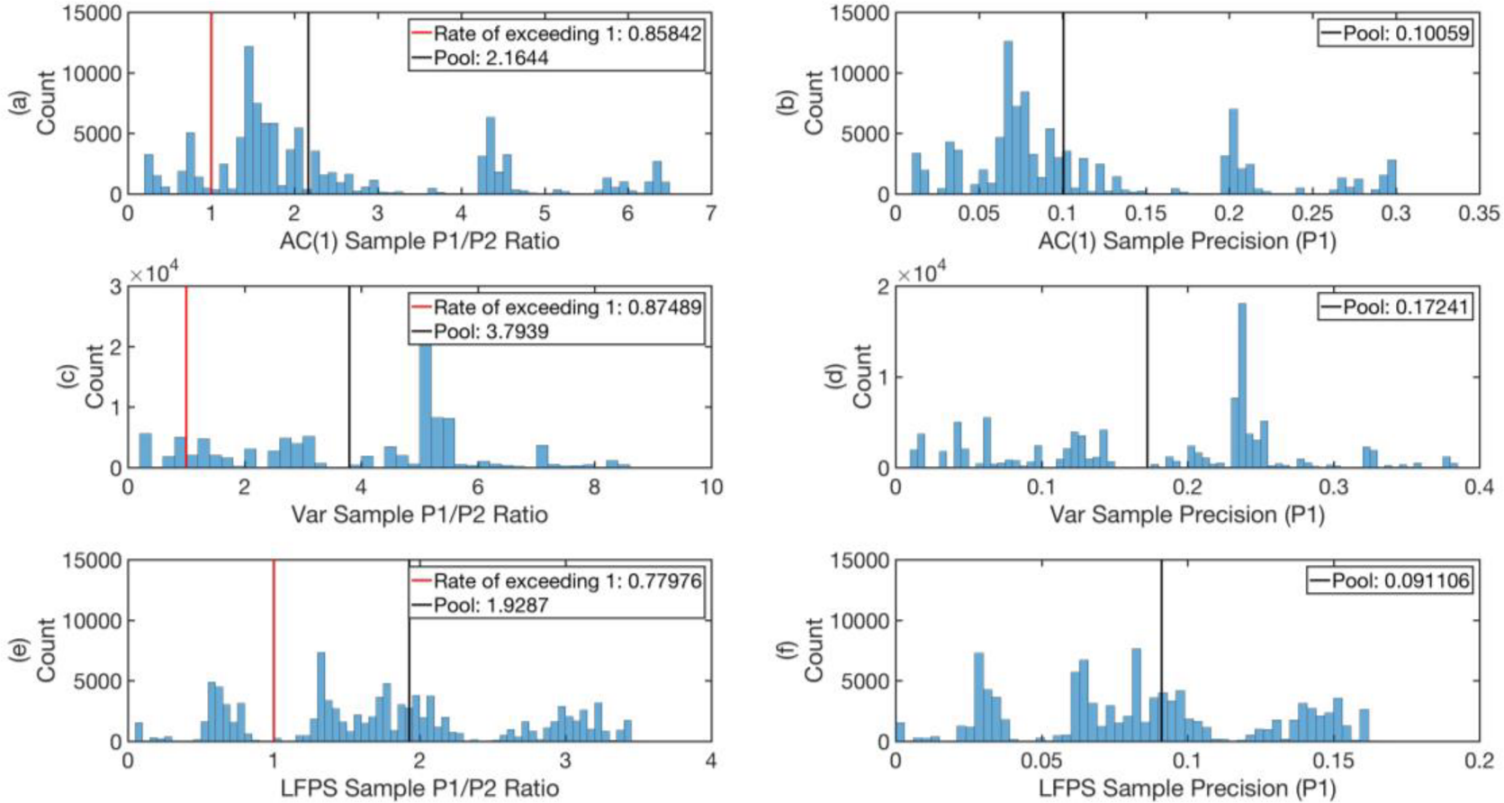
**The histograms of the ratios $\frac{P1}{P2}$ ((a), (c), and (e)) and precisions (*P***_**1**_**) ((b), (d), and (f)) of the 100,000 samples with 250 days trial period, for the indicators AC(1), Var, and LFPS respectively for the data set AUD-JPY from 1996 to 2004.** The red vertical lines mark $\frac{P1}{P2}=1$, and in the legends we give the proportion of samples with $\frac{P1}{P2}>1$ in the 100,000 samples as *rate of exceeding 1*. The pool values of $\frac{P1}{P2}$ and *P*_1_ are marked by black vertical lines.

**Fig 13:**
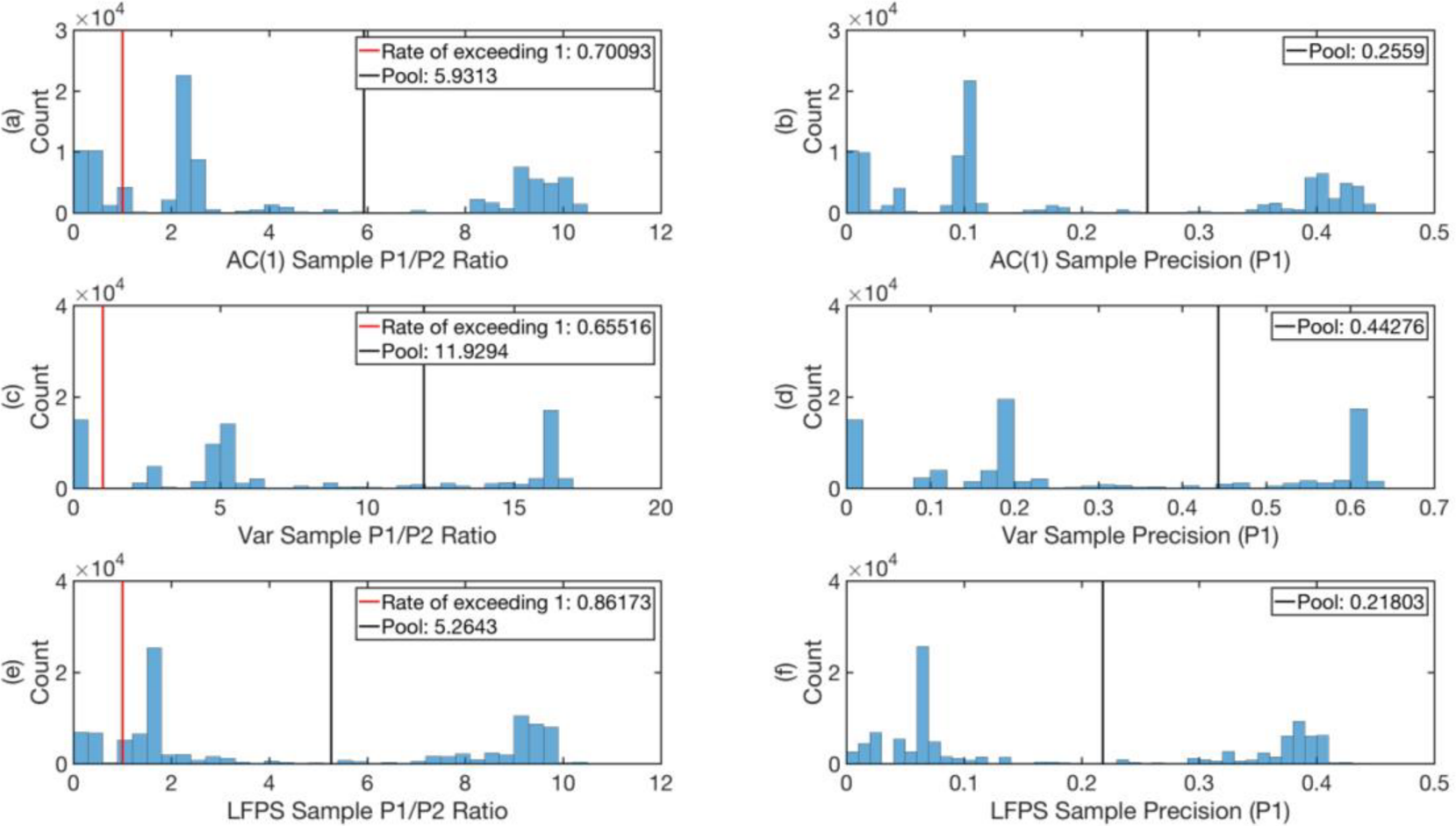
**The histograms of the ratios $\frac{P1}{P2}$ ((a), (c), and (e)) and precisions (*P***_**1**_**) ((b), (d), and (f)) of the 100,000 samples with 250 days trial period, for the indicators AC(1), Var, and LFPS respectively for the data set AUD-JPY from 2005 to 2010.** The red vertical lines mark $\frac{P1}{P2}=1$, and in the legends we give the proportion of samples with $\frac{P1}{P2}>1$ in the 100,000 samples as *rate of exceeding 1*. The pool values of $\frac{P1}{P2}$ and *P*_1_ are marked by black vertical lines.

**Fig 14:**
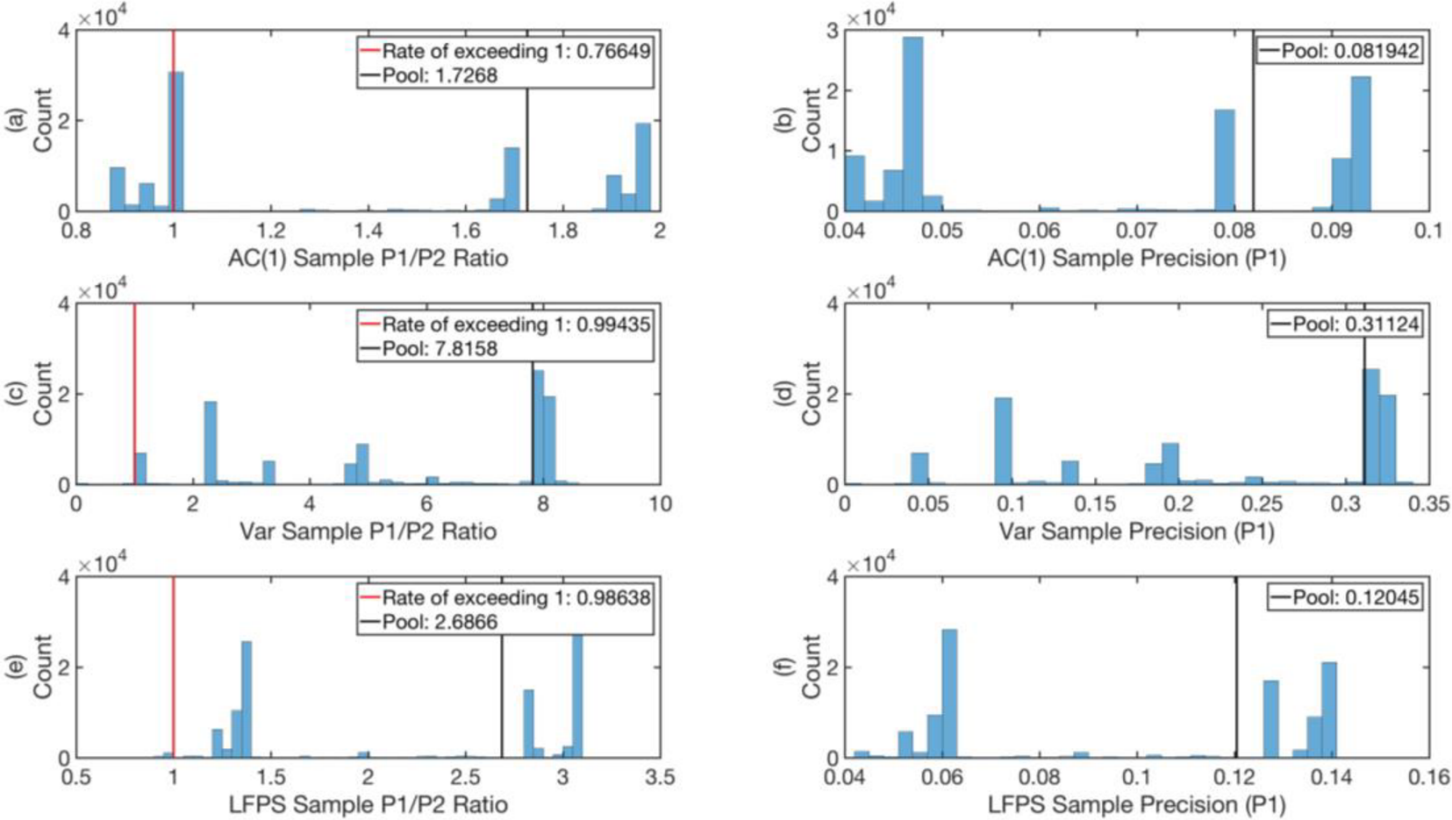
**The histograms of the ratios $\frac{P1}{P2}$ ((a), (c), and (e)) and precisions (*P***_**1**_**) ((b), (d), and (f)) of the 100,000 samples with 250 days trial period, for the indicators AC(1), Var, and LFPS respectively for the data set CHF-JPY.** The red vertical lines mark $\frac{P1}{P2}=1$, and in the legends we give the proportion of samples with $\frac{P1}{P2}>1$ in the 100,000 samples as *rate of exceeding 1*. The pool values of $\frac{P1}{P2}$ and *P*_1_ are marked by black vertical lines.

Indeed, as can be seen from **Figs 12–14** (See Text I in S1 Protocol for Matlab script), the pool ratio $\frac{P1}{P2}$ averaged over all times is greater than 1 for all indicators in all data sets. In the worst case, for AC(1) of CHF-JPY, this ratio is 1.73, whereas in the best case, for Var of AUD-JPY (2005–2010), the ratio is 11.93. These performances determine what we would have gotten from acting on the EWSs all the time for the three data sets.

Since it is customary for traders to test a new strategy over a finite time period before adopting it, we also tested the reliability of the EWSs over various shorter time periods. For example, to test the reliability of the EWSs on the scale of 250 trading days, we created a statistical ensemble of 250-trading-day time period with 100,000 random starting times. We then computed the histograms of $\frac{P1}{P2}$ and *P*_1_ over this ensemble, as shown in **Figs 12–14** for the data sets AUD-JPY from 1996 to 2004, AUD-JPY from 2005 to 2010, and CHF-JPY from 2008 to 2009 respectively. In the histograms of $\frac{P1}{P2}$, we highlighted $\frac{P1}{P2}=1$ with red vertical lines. These lines separate the samples with $\frac{P1}{P2}<1$ from the ones with $\frac{P1}{P2}>1$, whose proportions in the 100,000 samples are given by *rate of exceeding 1* in the legends. From **Figs 12–14**, we see that for the case of sampling with 250 trading days, the *rates of exceeding 1* for all indicators in all data sets are above 0.7, except for that of Var for AUD-JPY (2005–2010) in **Fig 13(C)**, which is 0.66. This implies that the EWSs are informative most of the time, and perform better at predicting large maximum spreads than just pure guessing. The expectations of $\frac{P1}{P2}$ for the ensembles are close to their pool values marked as black vertical lines in the limit of large sample size (100,000). These expectation values are even larger than 1, meaning that on average the EWSs carry significant information on predicting large maximum spreads. The histograms of *P*_1_ (**(b)**, **(d)**, and **(f)** of **Figs 12–14**) show the distributions of *precisions* of EWSs, with their pool values marked as black vertical lines. From these, we can see that most pool values are larger than 0.1, with only two exceptions in **Figs 12(F)** and **14(B)**. Note that in **Fig 14**, the bands are highly concentrated. This is because the CHF-JPY data set contains only 382 trading days, which is not large enough to sample many 250-trading-day windows with random starting time. In comparison, AUD-JPY (1996–2004) and AUD-JPY (2005–2010) include 2608 and 1311 trading days respectively, which is large enough for this test.

**Fig 15:**
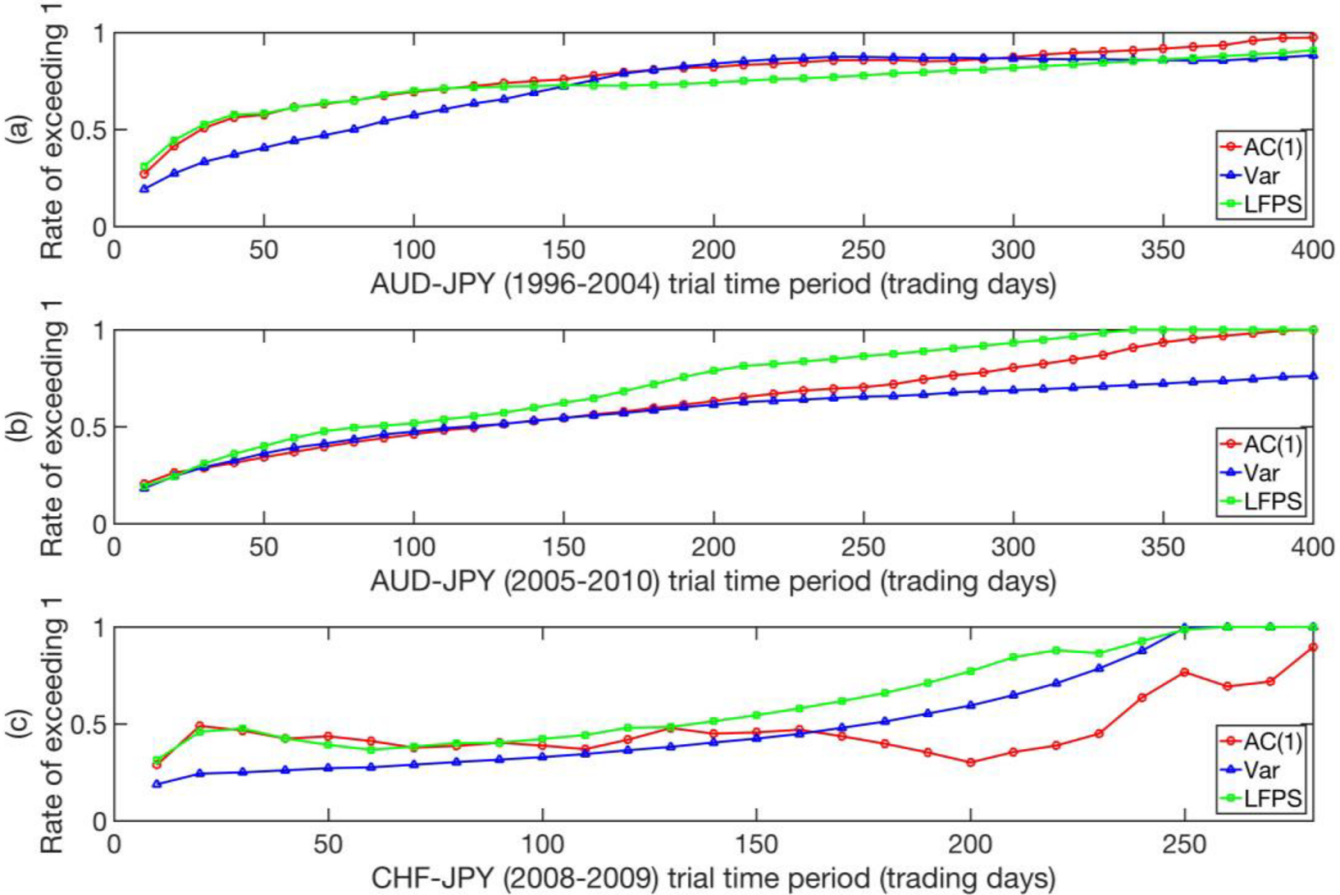
***Rates of exceeding 1* for the ratios $\frac{P1}{P2}$ with increasing trial time period, for the indicators AC(1), Var, and LFPS and data sets (a) AUD-JPY (1996–2004), (b) AUD-JPY (2005–2010), and (c) CHF-JPY (2008–2009).** Each data point is computed with 100,000 samples.

To see how the performances of EWSs measured by *rates of exceeding 1* change with varying trial time periods, we repeated the same sampling procedure with a growing time period starting from 10 trading days up to 400 trading days in steps of 10 trading days. The results are shown in **Fig 15** (See Text J in S1 Protocol for Matlab script). From **Fig 15(A)** and **15(B)**, we see that *rates of exceeding 1* increase monotonously with trial time period, and gradually approaches the upper bound of 1, except for Var in **Fig 15(B)**, which grows very slowly. This implies that in practice, the overall performances of EWSs are expected to improve if they are tested for a longer time. In **Fig 15(C)**, we tested up to 280 trading days for CHF-JPY since it only contains 382 trading days’ worth of data. From **Fig 15(C)** we see an odd trend in the AC(1) curve from 150 trading days onwards, which might be a result of the small size of the CHF-JPY data set, limiting the variation of the starting time of long-time-period samples.

#### Conditions for EWSs

We have shown in this paper that statistical significant detection of EWSs is very sensitive to (1) the intrinsic early warning period for each extreme event, (2) the frequency of data points in the time series, and (3) the choice of test statistic for which the EWSs would be statistically significant. If the intrinsic early warning period is too short or the data frequency too low, we might end up with an insignificant value for the Kendall-tau even if we have independent and reliable validation of the critical transition tested.

Working with the stringent Kendall-tau statistic represents a desire by the early warnings community to be strict with which events they can claim as critical transitions. The data frequency is frequently within our control: if the experimental method and cost permit, we can always collect more data points per unit time. However, the intrinsic early warning period, which is the period of time the complex system we study re-organizes and move endogenously towards the critical transition, is something that we may have little control over. Moreover, we have no theoretical justification that critical transitions of the same scale have similar intrinsic early warning periods. A large critical transition may thus be accompanied by a short early warning period, and we would then simply miss its early warnings.

#### The impact of accidental noise sequences

In this final subsection, we discuss how robust our conclusions are, when there is noise in the time series data. The first question we would ask is how likely it is for us to observe a statistically significant EWS that is due entirely to random noise. In some sense, this is also the easiest question to answer: the probability of a series of purely random noises producing an EWS that is statistically significant is given by the p value of our statistical test. In all our tests, which involve reshuffling the time series data to obtain a statistical ensemble of artificial data that has no serial correlation in time, this probability is at the level of less than 0.05, i.e. no more than 5% of the EWSs that we have identified can be due entirely to random noise.

The next question we might ask is how we can separate an accidental sequence of noises that is meaningless from an intrinsic trend that is meaningful. One might worry that these two cannot be disentangled when we use high-frequency data, especially when the time scale over which the critical transitions occur is short. We made clear in the **Effects of increasing time interval** subsection that (1) the typical time scale over which extreme movements of the FOREX market occur is around 15 minutes, which is already 2 orders of magnitude more than the time interval *T*_0_ we used in our analyses, and (2) intrinsic trends that preceded large exchange rate variations, which we call the early warning periods, lasted up to 3 days. Again, this time scale is very much larger than *T*_0_. There is thus no worries that fluctuations at the scale of *T*_0_ will impact the conclusions we arrived at, because for this to happen, we would need the fluctuation to be accidentally correlated over thousands to ten thousands of time steps, which is extremely unlikely.

The last question concerns more the correct identification of booms/crashes. This is a fair question to ask for a paper like ours, but is one that is extremely difficult to answer. In the stock market, there have been many attempts to define market crashes, but none of these definitions are universally accepted because they are not based on a mechanistic understanding of the market. In place of a rigorous definition, researchers have resorted to studying market crashes that are reported in the popular press. These are frequently the most pronounced crashes, and therefore are the least controversial. Many smaller crashes are likely to have been missed, because they are not picked up by financial news reporters.

In particular, with the advent of high-frequency algorithmic trading, flash crashes of the order of 10% in market value but lasting several minutes are not uncommon in major exchanges of the world. These are assumed to be due to glitches in the trading algorithms, but are poorly documented and studied. A similar problem plagues the FOREX market. Because of the shorter time scale on the FOREX market, one naturally expects many more booms and crashes in a given period of time. These events are rarely picked up by financial news reporters, so we do not even have a curated list of the most uncontroversial events to work with. This is why in this paper we used the 95^th^ percentile set of the maximum spread as a proxy for booms and crashes, because there is no ground truth we can obtain by alternative means.

## Supporting information

S1 Protocol Matlab protocols. (DOCX)

S1 Appendix Tables in sensitivity analyses. (DOCX)
