# Supplementary material for: How one might miss early warning signals of critical transitions in time series data: A systematic study of two major currency pairs
Source: PLoS One. 2018 Mar 14;13(3):e0191439. doi: 10.1371/journal.pone.0191439 (PMC5851542; doi:10.1371/journal.pone.0191439)
Supplement: S1 Protocol — (DOCX) [file pone.0191439.s001.docx]

How One Might Miss Early Warning Signals of Critical Transitions in Time Series Data: A Systematic Study of Two Major Currency Pairs

S1 Support Information: Matlab protocols

Haoyu Wen,^1,2*^ Massimo Pica Ciamarra,^1,2^  Siew Ann Cheong^1,2^

^1^ Division of Physics and Applied Physics, School of Physical and Mathematical Sciences, Nanyang Technological University, Singapore

^2^ Complexity Institute, Nanyang Technological University, Singapore

* Corresponding author

Email: [s160049@e.ntu.edu.sg](mailto:s160049@e.ntu.edu.sg) (H. W)

**List of contents**

- The Protocol
- Text A. Matlab script for converting original data into fixed time intervals (15s or 30s)
- Text B. Matlab script for applying Gaussian de-trending to the time series data with fixed time interval
- Text C. Matlab script for computing EWSs from AC(1)
- Text D. Matlab script for computing EWSs from Var
- Text E. Matlab script for computing EWSs from LFPS
- Text F. Matlab script for applying criteria of historical P value of endpoints on EWSs
- Text G. Matlab script for overlapping EWSs from all three indicators
- Text H. Matlab script for computing DR and SP
- Text I. Matlab script for reliability analysis (Figs 12 - 14)
- Text J. Matlab script for reliability analysis (Fig 15)

**The Protocol.**

1) Use Matlab script (Text A) to convert original data into fixed time intervals. End product: *newM* (n-by-2 matrix)

Tunable parameters: *STL*, *TTL*

2) Use Matlab script (Text B) to apply Gaussian de-trending to newM.

End product: *Residue* (n-by-2 matrix)

Tunable parameters: *wid*

3) Find EWSs from AC(1), Var, and LFPS by Texts C, D, and E, respectively.

End product: *signal* (n-by-8 matrix)

Tunable parameters: *samplesize, window,* *R_window, R_step, incre, inner_incre, Threshold_Pvalue_ken, Threshold_Phist_end*

4) Apply criteria of historical P value of endpoints on EWSs such that only the ones with low enough historical P value are selected to be significant EWSs. (Text F)

End product: *signal*

Tunable parameters: *Phist*

5) Obtain concurrent EWSs using Text G

End product: *overlap_signal*

Tunable parameters: *Endpoint_Phist*

6) Compute DR and SP using Text H multiple times, each time for one EWS data file

End product:

*Disc_rate_10pct_vshist* (DR10)

*Disc_rate_5pct_vshist* (DR5)

*Component_10pct_vssigs* (SP10)

*Component_5pct_vssigs* (SP5)

Tunable parameters: the EWS data file, *DT_max, R_step*

7) Reliability analyses (Texts I and J)

End product: reliability analyses figures

Tunable parameters (Both I and J): *TI, TW, topxpct, Duration_EWS, P_kendall_max, P_endpoint_max, N_samples, R_step, Size_Sample_days*(Text J only)

**Text A. Matlab script for converting original data into fixed time intervals (15s or 30s)**

load('CHFJPY_08to09.mat')

Mat = D_08_09; % i.e. the original data matrix, AUDJPY_05to10 bid price second col

% first remove NaN entries

Mat(isnan(Mat(:,2)), :) = [];

% then remove entries with unchanged prices for more than 10 ticks

UnchP = diff(Mat(:,2)) == 0; % 1 for unchanged prices, 0 for changed prices

lenP = length(UnchP);

pool = 0;

alarm = 0;

Remove = zeros(lenP, 1);

for i = 1:lenP

if UnchP(i) == 1

pool = pool + 1;

else % i.e. UnchP(i) == 0

if alarm == 0

pool = 0;

continue

else % i.e. alarm == 1, which means pool >= 10 already

Remove(i-pool+1:i-1) = Remove(i-pool+1:i-1) + 1;

alarm = 0;

pool = 0;

end

end

if pool >= 10

alarm = 1;

end

end

Mat(Remove == 1, :) = [];

% now continue

oldT = Mat(:,1);

len = length(oldT);

newM = zeros(len, 2); % create the new data matrix

newM(1,:) = Mat(1,:); % start with the first row

STL = 30/86400; % the standard ticking length, 15 s here

TTL = 90/86400; % the tolerable ticking length, 90s here

sum = 0;

r = 2; % the index of the row to add value to newM

for i = 1:len-1

interval = oldT(i+1) - oldT(i); % the ticking length in old matrix

sum = sum + interval; % add up for decision makings later

if sum > STL

if sum < 2*STL % i.e. a standard case

newM(r, :) = [newM(r-1,1)+STL, Mat(i, 2)];

sum = sum - STL;

r = r + 1;

else % i.e. sum > 2*STL

if sum < TTL % within tolerance

% then check how long the interval is and thus assign

% proper numbers of sets of same values to newM

num_int = floor(sum/STL);

for j = 1:num_int

temp_T = newM(r-1,1) + STL;

newM(r, :) = [temp_T Mat(i,2)];

r = r+1;

sum = sum - STL;

end

else % sum is more than TTL

newM(r,:) = Mat(i,:);

r = r+1;

sum = 0;

end

end

end

end

% then remove the redundant zeros

for k = 1:len

if newM(k,1) == 0

if newM(k+1,1) == 0 % double check

newM(k:len,:) = [];

break

end

end

end

save('CHFJPY_standard_30s_90s.mat', 'newM')

**Text B. Matlab script for applying Gaussian de-trending to the time series data with fixed time interval**

load('CHFJPY_standard_30s_90s.mat') % the data matrix with uniform ticking length

vec = newM(:,2);%(4000000:4001000,2); % the price series

wid = 48; % the bandwidth

halfWidth = wid / 2;

gaussFilter = gausswin(wid);

gaussFilter = gaussFilter / sum(gaussFilter); % Normalize.

smoothedVector = conv(vec, gaussFilter);

smoothed = smoothedVector(halfWidth:end-halfWidth); %change here

diffvec = vec - smoothed; % the residue series

T = newM(:,1);

Residue = [T diffvec];

save('CHFJPY_standard_30s_90s_gaussian_residue_nby2_wid48.mat', 'Residue')

**Text C. Matlab script for computing EWSs from AC(1)**

**Text C(1). Main script**

load('AUDJPY_standard_15s_90s_gaussian_residue_nby2_wid150.mat')

% key parameters initialization

samplesize = 1000; % how many samples for P value test

window = 84; % the length of LFPS sequence to test kendall and Pvalue for

R_window = 114; % Residue(100*140 + 50200, 1) - Residue(50000, 1) = 2.73 days

R_step = 38;

incre = 1; % the number of entries of indicators each time passes when checking

inner_incre = 1; % number of indicators to pass after confirmed EWS

Threshold_Pvalue_ken = 0.05; % original set 0.05

Threshold_Phist_end = 0.08;

bandwidth = window; % for the Gaussian kernel smooth

% compute indicators across time first

S = size(Residue);

full_len = S(1);

No_indicators = floor((full_len-2*R_window)/R_step);

Indicators_full = zeros(No_indicators, 1);

for i = 1:No_indicators

Residue_per_indicator = Residue((1+(i-1)*R_step):(R_window+(i-1)*R_step), 2);

acf = autocorr(Residue_per_indicator, 1);

Indicators_full(i) = acf(2);

end

% gather kendall tau, gradient, and endpoint throughout the time first for

% historical P values of the three

Kendall_all = zeros(No_indicators-2*window, 1);

Endpoint_all = Kendall_all;

Y = (1:window)';

for j = 1:(No_indicators-2*window)

Indicator_window = Indicators_full(j:(j+window-1));

Kendall_all(j) = corr(Indicator_window, Y, 'type', 'Kendall');

smoothed = gaussian_kernel(Indicator_window, bandwidth);

Endpoint_all(j) = smoothed(window); % last entry of Gaussian smoothed curve as endpoint

end

fprintf('Full historical data finished \n')

tic

entry = 1; % where the examine is up to, index in Residue

signal = zeros(10000, 8); % to store the signals and their properties

sig_row = 1; % row to fill in next in signal

while entry <= (full_len - 2*R_window - window*R_step)

Residue_part = Residue(entry:(entry+R_window+window*R_step), :); %??!!

B = 1;

Size = size(Residue_part);

N = Size(1);

All_SP = (1:N)';

bootstrap_AR1_v8

Kendall = Kendalls(1);

Endpoint = Endpoints(1);

Num_end = 0;

for i = 1:length(Kendall_all)

if Endpoint_all(i) > Endpoint

Num_end = Num_end + 1;

end

end

P_hist_end = Num_end/length(Kendall_all);

if P_hist_end > Threshold_Phist_end

entry = entry + incre*R_step;

continue

end

% if the loop reaches here, start computing P value

B = samplesize;

rng('shuffle')

All_SP = zeros(N, B);

for ii = 1:B

All_SP(:, ii) = datasample((1:N)', N,'Replace', false);

end

bootstrap_AR1_v7

% test for P value, of Kendall, Gradient, and Endpoint

Num_ken = 0;

for i = 1:B

if Kendalls(i) > Kendall

Num_ken = Num_ken + 1;

end

end

P_ken = Num_ken/B;

if P_ken > Threshold_Pvalue_ken % ------------HERE!------------

entry = entry + incre*R_step;

continue

end

% fill in the details of this EWS

signal(sig_row, 1) = Residue(entry, 1); % signal start time

signal(sig_row, 2) = Residue(entry+R_window+window*R_step, 1); % signal end time

signal(sig_row, 3) = entry; % signal start index

signal(sig_row, 4) = entry+R_window+window*R_step; % signal end index

signal(sig_row, 5) = Kendall; % signal kendall

signal(sig_row, 6) = P_ken;

signal(sig_row, 7) = Endpoint; % representing level of indicator (high/low)

signal(sig_row, 8) = P_hist_end;

% finally, update variables and move on to next iteration

sig_row = sig_row + 1;

entry = entry+inner_incre*R_step;

run_time = toc;

finished_percent = (entry/full_len)*100;

time_past = run_time/60;

time_left = ((full_len - entry)/entry)*time_past;

progress_time = [finished_percent time_past time_left];

disp(progress_time)

end

toc

**Text C(2). Script: bootstrap_AR1_v7**

Kendalls = zeros(B, 1); % to store kendalls

Endpoints = Kendalls;

for j = 1:B

ind = All_SP(:, j); % the index

C_2 = Residue_part(:,2);

% now sample is constructed, get AR next

Target = C_2(ind);

ARparameters = zeros(1,window)'; % to store the AR parameter for each interval

for i = 1:window

submat = Target((1+i*R_step):((R_window + i*R_step))); % the rolling window

acf = autocorr(submat, 1);

ARparameters(i) = acf(2);

end

% now we get ARparameters (n-by-1)

% next, get Kendall

Kendalls(j) = corr(ARparameters, Y, 'type', 'Kendall');

smoothed = gaussian_kernel(ARparameters, bandwidth);

Endpoints(j) = smoothed(window);

end

**Text C(3). Script: bootstrap_AR1_v7**

Kendalls = zeros(B, 1); % to store kendalls

for j = 1:B

ind = All_SP(:, j); % the index

C_2 = Residue_part(:,2);

% now sample is constructed, get AR next

Target = C_2(ind);

ARparameters = zeros(1,window)'; % to store the AR parameter for each interval

for i = 1:window

submat = Target((1+i*R_step):((R_window + i*R_step))); % the rolling window

acf = autocorr(submat, 1);

ARparameters(i) = acf(2);

end

% now we get ARparameters (n-by-1)

% next, get Kendall

Kendalls(j) = corr(ARparameters, Y, 'type', 'Kendall');

end

**Text D.** **Matlab script for computing EWSs from Var**

**Text D(1). Main script**

load('AUDJPY_standard_15s_90s_gaussian_residue_nby2_wid150.mat')

% key parameters initialization

samplesize = 1000; % how many samples for P value test

window = 84; % the length of LFPS sequence to test kendall and Pvalue for

R_window = 114; % Residue(100*140 + 50200, 1) - Residue(50000, 1) = 2.73 days

R_step = 38;

incre = 1; % the number of entries of indicators each time passes when checking

inner_incre = 1; % number of indicators to pass after confirmed EWS

Threshold_Pvalue_ken = 0.05; % original set 0.05

Threshold_Phist_end = 0.08;

bandwidth = window; % for the Gaussian kernel smooth

% compute indicators across time first

S = size(Residue);

full_len = S(1);

No_indicators = floor((full_len-2*R_window)/R_step);

Indicators_full = zeros(No_indicators, 1);

for i = 1:No_indicators

Residue_per_indicator = Residue((1+(i-1)*R_step):(R_window+(i-1)*R_step), 2);

Indicators_full(i) = var(Residue_per_indicator);

end

% gather kendall tau, gradient, and endpoint throughout the time first for

% historical P values of the three

Kendall_all = zeros(No_indicators-2*window, 1);

Endpoint_all = Kendall_all;

Y = (1:window)';

for j = 1:(No_indicators-2*window)

Indicator_window = Indicators_full(j:(j+window-1));

Kendall_all(j) = corr(Indicator_window, Y, 'type', 'Kendall');

smoothed = gaussian_kernel(Indicator_window, bandwidth);

Endpoint_all(j) = smoothed(window); % last entry of Gaussian smoothed curve as endpoint

end

fprintf('Full historical data finished \n')

tic

entry = 1; % where the examine is up to, index in Residue

signal = zeros(10000, 8); % to store the signals and their properties

sig_row = 1; % row to fill in next in signal

while entry <= (full_len - 2*R_window - window*R_step)

Residue_part = Residue(entry:(entry+R_window+window*R_step), :); %??!!

B = 1;

Size = size(Residue_part);

N = Size(1);

All_SP = (1:N)';

bootstrap_Var_v8

Kendall = Kendalls(1);

Endpoint = Endpoints(1);

Num_end = 0;

for i = 1:length(Kendall_all)

if Endpoint_all(i) > Endpoint

Num_end = Num_end + 1;

end

end

P_hist_end = Num_end/length(Kendall_all);

if P_hist_end > Threshold_Phist_end

entry = entry + incre*R_step;

continue

end

% if the loop reaches here, start computing P value

B = samplesize;

rng('shuffle')

All_SP = zeros(N, B);

for ii = 1:B

All_SP(:, ii) = datasample((1:N)', N,'Replace', false);

end

bootstrap_Var_v7

% test for P value, of Kendall, Gradient, and Endpoint

Num_ken = 0;

for i = 1:B

if Kendalls(i) > Kendall

Num_ken = Num_ken + 1;

end

end

P_ken = Num_ken/B;

if P_ken > Threshold_Pvalue_ken % ------------HERE!------------

entry = entry + incre*R_step;

continue

end

% fill in the details of this EWS

signal(sig_row, 1) = Residue(entry, 1); % signal start time

signal(sig_row, 2) = Residue(entry+R_window+window*R_step, 1); % signal end time

signal(sig_row, 3) = entry; % signal start index

signal(sig_row, 4) = entry+R_window+window*R_step; % signal end index

signal(sig_row, 5) = Kendall; % signal kendall

signal(sig_row, 6) = P_ken;

signal(sig_row, 7) = Endpoint; % representing level of indicator (high/low)

signal(sig_row, 8) = P_hist_end;

% finally, update variables and move on to next iteration

sig_row = sig_row + 1;

entry = entry+inner_incre*R_step;

run_time = toc;

finished_percent = (entry/full_len)*100;

time_past = run_time/60;

time_left = ((full_len - entry)/entry)*time_past;

progress_time = [finished_percent time_past time_left];

disp(progress_time)

end

toc

**Text D(2). Script: bootstrap_Var_v8**

Kendalls = zeros(B, 1); % to store kendalls

Endpoints = Kendalls;

for j = 1:B

ind = All_SP(:, j); % the index

C_2 = Residue_part(:,2);

% now sample is constructed, get AR next

Target = C_2(ind);

Variances = zeros(1,window)'; % to store the AR parameter for each interval

for i = 1:window

submat = Target((1+i*R_step):((R_window + i*R_step))); % the rolling window

Variances(i) = var(submat);

end

% now we get ARparameters (n-by-1)

% next, get Kendall

Kendalls(j) = corr(Variances, Y, 'type', 'Kendall');

smoothed = gaussian_kernel(Variances, bandwidth);

Endpoints(j) = smoothed(window);

end

**Text D(3).** **Script: bootstrap_Var_v7**

Kendalls = zeros(B, 1); % to store kendalls

for j = 1:B

ind = All_SP(:, j); % the index

C_2 = Residue_part(:,2);

% now sample is constructed, get AR next

Target = C_2(ind);

Variances = zeros(1,window)'; % to store the AR parameter for each interval

for i = 1:window

submat = Target((1+i*R_step):((R_window + i*R_step))); % the rolling window

Variances(i) = var(submat);

end

% now we get ARparameters (n-by-1)

% next, get Kendall

Kendalls(j) = corr(Variances, Y, 'type', 'Kendall');

end

**Text E.** **Matlab script for computing EWSs from LFPS**

**Text E(1). Main script**

load('AUDJPY_standard_15s_90s_gaussian_residue_nby2_wid150.mat')

% key parameters initialization

samplesize = 1000; % how many samples for P value test

window = 84; % the length of LFPS sequence to test kendall and Pvalue for

R_window = 114; % Residue(100*140 + 50200, 1) - Residue(50000, 1) = 2.73 days

R_step = 38;

incre = 1; % the number of entries of indicators each time passes when checking

inner_incre = 1; % number of indicators to pass after confirmed EWS

Threshold_Pvalue_ken = 0.05; % original set 0.05

Threshold_Phist_end = 0.08;

bandwidth = window; % for the Gaussian kernel smooth

Crit_num = 0.26;

Criteria = floor(R_window*Crit_num); % the criteria for low frequency

% compute indicators across time first

S = size(Residue);

full_len = S(1);

No_indicators = floor((full_len-2*R_window)/R_step);

Indicators_full = zeros(No_indicators, 1);

for i = 1:No_indicators

Residue_per_indicator = Residue((1+(i-1)*R_step):(R_window+(i-1)*R_step), 2);

power = (abs(fft(Residue_per_indicator))).^2;

power = power./(sum(power)); % normalize

lowfreqpower = power(1:Criteria); % the low frequency part

Indicators_full(i) = sum(lowfreqpower);

end

% gather kendall tau, gradient, and endpoint throughout the time first for

% historical P values of the three

Kendall_all = zeros(No_indicators-2*window, 1);

Endpoint_all = Kendall_all;

Y = (1:window)';

for j = 1:(No_indicators-2*window)

Indicator_window = Indicators_full(j:(j+window-1));

Kendall_all(j) = corr(Indicator_window, Y, 'type', 'Kendall');

smoothed = gaussian_kernel(Indicator_window, bandwidth);

Endpoint_all(j) = smoothed(window); % last entry of Gaussian smoothed curve as endpoint

end

fprintf('Full historical data finished \n')

tic

entry = 1; % where the examine is up to, index in Residue

signal = zeros(10000, 8); % to store the signals and their properties

sig_row = 1; % row to fill in next in signal

while entry <= (full_len - 2*R_window - window*R_step)

Residue_part = Residue(entry:(entry+R_window+window*R_step), :); %??!!

B = 1;

Size = size(Residue_part);

N = Size(1);

All_SP = (1:N)';

bootstrap_LFPS_v8

Kendall = Kendalls(1);

Endpoint = Endpoints(1);

Num_end = 0;

for i = 1:length(Kendall_all)

if Endpoint_all(i) > Endpoint

Num_end = Num_end + 1;

end

end

P_hist_end = Num_end/length(Kendall_all);

if P_hist_end > Threshold_Phist_end

entry = entry + incre*R_step;

continue

end

% if the loop reaches here, start computing P value

B = samplesize;

rng('shuffle')

All_SP = zeros(N, B);

for ii = 1:B

All_SP(:, ii) = datasample((1:N)', N,'Replace', false);

end

bootstrap_LFPS_v7

% test for P value, of Kendall, Gradient, and Endpoint

Num_ken = 0;

for i = 1:B

if Kendalls(i) > Kendall

Num_ken = Num_ken + 1;

end

end

P_ken = Num_ken/B;

if P_ken > Threshold_Pvalue_ken % ------------HERE!------------

entry = entry + incre*R_step;

continue

end

% fill in the details of this EWS

signal(sig_row, 1) = Residue(entry, 1); % signal start time

signal(sig_row, 2) = Residue(entry+R_window+window*R_step, 1); % signal end time

signal(sig_row, 3) = entry; % signal start index

signal(sig_row, 4) = entry+R_window+window*R_step; % signal end index

signal(sig_row, 5) = Kendall; % signal kendall

signal(sig_row, 6) = P_ken;

signal(sig_row, 7) = Endpoint; % representing level of indicator (high/low)

signal(sig_row, 8) = P_hist_end;

% finally, update variables and move on to next iteration

sig_row = sig_row + 1;

entry = entry+inner_incre*R_step;

run_time = toc;

finished_percent = (entry/full_len)*100;

time_past = run_time/60;

time_left = ((full_len - entry)/entry)*time_past;

progress_time = [finished_percent time_past time_left];

disp(progress_time)

end

toc

**Text E(2). Script: bootstrap_LFPS_v8**

Kendalls = zeros(B, 1); % to store kendalls

Endpoints = Kendalls;

for j = 1:B

ind = All_SP(:, j); % the index

C_2 = Residue_part(:,2);

% now sample is constructed, get AR next

Target = C_2(ind);

Variances = zeros(1,window)'; % to store the AR parameter for each interval

for i = 1:window

submat = Target((1+i*R_step):((R_window + i*R_step))); % the rolling window

Variances(i) = var(submat);

end

% now we get ARparameters (n-by-1)

% next, get Kendall

Kendalls(j) = corr(Variances, Y, 'type', 'Kendall');

smoothed = gaussian_kernel(Variances, bandwidth);

Endpoints(j) = smoothed(window);

end

**Text E(3). Script: bootstrap_LFPS_v7**

Kendalls = zeros(B, 1); % to store kendalls

for j = 1:B

ind = All_SP(:, j); % the index

C_2 = Residue_part(:,2);

% now sample is constructed, get AR next

Target = C_2(ind);

Variances = zeros(1,window)'; % to store the AR parameter for each interval

for i = 1:window

submat = Target((1+i*R_step):((R_window + i*R_step))); % the rolling window

Variances(i) = var(submat);

end

% now we get ARparameters (n-by-1)

% next, get Kendall

Kendalls(j) = corr(Variances, Y, 'type', 'Kendall');

end

**Text F. Matlab script for applying criteria of historical P value of endpoints on EWSs**

len_sig = size(signal,1);

Phist = 0.025

% remove zeros

for i = 1:len_sig

if signal(i, 1) == 0

signal(i:len_sig, :) = [];

break

end

end

% remove NaNs if any

signal(isnan(signal(:,3)), :) = [];

len_sig = size(signal,1);

counter = 0;

for i = 1:len_sig

if signal(i - counter, 8) > Phist % filter: endpoint Phist

signal(i - counter, :) = [];

counter = counter + 1;

end

end

**Text G. Matlab script for overlapping EWSs from all three indicators**

load('CHFJPY_standard_30s_90s_gaussian_residue_nby2_wid48.mat')

T = Residue(:,1); % the time axis

full_len = length(T);

signal_summary = zeros(full_len, 5);

signal_summary(:, 1) = T;

% 1st: T, 2nd: AR, 3rd: Var, 4th: LFPS, 5th: overlap signal

% the signals (col 2 to 4) are represented by numbers of EWS appearance

% within the time indicated by T

Endpoint_Phist = 0.06;

load('V8_GF_CHFJPY_AR_30s_90s_gauswid48_Rwin150_Rst50_win84_inc1_ininc1_samp1000_band84_Phist0pt08.mat')

% signal picker starts

size_sig = size(signal);

len_sig = size_sig(1);

% remove zeros

counter = 0;

for i = 1:len_sig

if signal(i - counter,1) == 0

signal(i - counter, :) = [];

counter = counter + 1;

end

end

% remove NaNs if any

check = isnan(signal(:,3));

signal(check, :) = [];

size_sig = size(signal);

len_sig = size_sig(1);

counter = 0;

for i = 1:len_sig

if signal(i - counter, 8) > Endpoint_Phist % flter3: endpoint

signal(i - counter, :) = [];

counter = counter + 1;

end

end

AR_signals = signal;

AR_sig_len = length(signal);

% signal picker ends

load('V8_GF_CHFJPY_Var_30s_90s_gauswid48_Rwin150_Rst50_win84_inc1_ininc1_samp1000_band84_Phist0pt08.mat')

% signal picker starts

size_sig = size(signal);

len_sig = size_sig(1);

% remove zeros

counter = 0;

for i = 1:len_sig

if signal(i - counter,1) == 0

signal(i - counter, :) = [];

counter = counter + 1;

end

end

% remove NaNs if any

check = isnan(signal(:,3));

signal(check, :) = [];

size_sig = size(signal);

len_sig = size_sig(1);

counter = 0;

for i = 1:len_sig

if signal(i - counter, 8) > Endpoint_Phist % flter3: endpoint

signal(i - counter, :) = [];

counter = counter + 1;

end

end

Var_signals = signal;

Var_sig_len = length(signal);

% signal picker ends

load('V8_GF_CHFJPY_LFPS_Crit24_30s_90s_gauswid48_Rwin150_Rst50_win84_inc1_ininc1_samp1000_band84_Phist0pt08.mat')

% signal picker starts

size_sig = size(signal);

len_sig = size_sig(1);

% remove zeros

counter = 0;

for i = 1:len_sig

if signal(i - counter,1) == 0

signal(i - counter, :) = [];

counter = counter + 1;

end

end

% remove NaNs if any

check = isnan(signal(:,3));

signal(check, :) = [];

size_sig = size(signal);

len_sig = size_sig(1);

counter = 0;

for i = 1:len_sig

if signal(i - counter, 8) > Endpoint_Phist % flter3: endpoint

signal(i - counter, :) = [];

counter = counter + 1;

end

end

LFPS_signals = signal;

LFPS_sig_len = length(signal);

% signal picker ends

% start to count and add into col 2 to 4 in signal_summary

for i = 1:AR_sig_len

signal_summary(AR_signals(i, 3):(AR_signals(i, 4)-1), 2) = signal_summary(AR_signals(i, 3):(AR_signals(i, 4)-1), 2) + 1;

end

for i = 1:Var_sig_len

signal_summary(Var_signals(i, 3):Var_signals(i, 4)-1, 3) = signal_summary(Var_signals(i, 3):Var_signals(i, 4)-1, 3) + 1;

end

for i = 1:LFPS_sig_len

signal_summary(LFPS_signals(i, 3):LFPS_signals(i, 4)-1, 4) = signal_summary(LFPS_signals(i, 3):LFPS_signals(i, 4)-1, 4) + 1;

end

% now col 2 to 4 is fill, start filling col 5

% the rule: if all col 2 to 4 is filled with >= 1 value, then col 5 fill

% with 1, otherwise 0. 1 means consistent overlap signal of all 3 present

for i = 1:full_len

for j = 2:4

if signal_summary(i, j) >= 1

signal_summary(i, j) = 1; % 1 for active, 0 for not

end

end

signal_summary(i, 5) = signal_summary(i, 2) + signal_summary(i, 3) + signal_summary(i, 4); % number of indicators active

end

% now signal_summary is ready

signal_points = find(signal_summary(:, 5) >= 3);

len_points = length(signal_points);

overlap_signal_ind = zeros(1000, 2);

overlap_signal_ind(1,1) = signal_points(1);

row = 1;

for i = 2:len_points

if signal_points(i) ~= signal_points(i-1) + 1

overlap_signal_ind(row,2) = signal_points(i - 1);

row = row + 1;

overlap_signal_ind(row,1) = signal_points(i);

end

end

overlap_signal_ind(row,2) = signal_points(end);

% remove zeros

counter = 0;

for i = 1:1000

if overlap_signal_ind(i - counter,1) == 0

overlap_signal_ind(i - counter, :) = [];

counter = counter + 1;

end

end

overlap_signal = [T(overlap_signal_ind(:,1)) T(overlap_signal_ind(:,2))];

**Text H. Matlab script for computing DR and SP**

load('V8_G7_AUDJPY_96to05_AR_30s_90s_gauswid76_Rwin126_Rst42_win86_inc1_ininc1_samp1000_band86_Phist0pt08.mat')

size_sig = size(signal);

len_sig = size_sig(1);

% remove zeros

counter = 0;

for i = 1:len_sig

if signal(i - counter,1) == 0

signal(i - counter, :) = [];

counter = counter + 1;

end

end

% remove NaNs if any

check = isnan(signal(:,3));

signal(check, :) = [];

%return

size_sig = size(signal);

len_sig = size_sig(1);

counter = 0;

Endpoint_Phist = 0.08; % ------------ chage here accordingly! ------------

for i = 1:len_sig

if signal(i - counter, 8) > Endpoint_Phist % flter: endpoint Phist

signal(i - counter, :) = [];

counter = counter + 1;

end

end

% new_signal_picker_v8 ends

%load('AUDJPY_96to05_standard_60s_240s.mat') % corresponding price time series, newM ------ change ------

load('AUDJPY_96to05_standard_45s_180s.mat')

%load('AUDJPY_96to05_standard_30s_90s.mat')

DTmax = floor(0.5*24*60*60/45); % the maximum Delta T within which T0 is found, 3days ------ /time interval ------

S = size(signal);

new_signal = zeros(S(1), S(2)+3);

new_signal(:, 1:S(2)) = signal;

len_newM = length(newM);

% new_signal: appended 1st col T0 index, 2nd col T0 time diff, 3rd col Return %

for i = 1:S(1)

if new_signal(i,4)+DTmax > len_newM

break

end

Price = newM(new_signal(i,4):(new_signal(i,4)+DTmax), :);

Price_diff = Price;

Price_diff(:,2) = Price(:,2) - Price(1,2);

[MaxP, MaxI] = max(abs(Price_diff(:,2))); % the maximum of abs(Price) and the index

new_signal(i, S(2)+1) = MaxI + signal(i,4); % T0 index

new_signal(i, S(2)+2) = Price(MaxI, 1) - Price(1, 1); % T0 time diff

new_signal(i, S(2)+3) = 100*(Price(MaxI,2) - Price(1, 2))/Price(1, 2); % Return %

end

% ------------- construct historical R vs T0 data -------------------------

% using rolling window of length DTmax and rolling step of 40

R_step = 28; % ----------------------- change here! -----------------------

len_hist = floor((length(newM)-DTmax)/R_step);

RvsT_hist = zeros(len_hist, 4);

for i = 1:len_hist

RvsT_hist(i, 1) = 1+i*R_step; % starting index of the rolling window

Price = newM(RvsT_hist(i, 1):(RvsT_hist(i, 1)+DTmax), :);

Price_diff = Price;

Price_diff(:,2) = Price(:,2) - Price(1,2);

[MaxP, MaxI] = max(abs(Price_diff(:,2))); % the maximum of abs(Price) and the index

RvsT_hist(i, 2) = MaxI + RvsT_hist(i, 1); % T0 index

RvsT_hist(i, 3) = Price(MaxI, 1) - Price(1, 1); % T0 time diff

RvsT_hist(i, 4) = 100*(Price(MaxI,2) - Price(1, 2))/Price(1, 2); % Return %

end

%scatter(RvsT_hist(:, 3), RvsT_hist(:, 4))

%h_hist = histogram(RvsT_hist(:, 4));

%h_hist.BinWidth = 0.2;

%xlim([-8 8])

% construct level lines of top 1%, 5%, and 10% of Return

abs_Rhist = sort(abs(RvsT_hist(:, 4)), 'descend');

R_top5pct = abs_Rhist(floor(len_hist*0.05));

R_top10pct = abs_Rhist(floor(len_hist*0.1));

R_top30pct = abs_Rhist(floor(len_hist*0.3));

% ------------- construct historical R vs T0 data done --------------------

% compare new_signal with historical data

Returns = new_signal(:, S(2)+3);

N_10pct = sum(abs(Returns) > R_top10pct); % number of signals that exceed 10 percentile extremeness

Disc_rate_10pct_vshist = N_10pct/(floor(length(RvsT_hist)*0.1))*100; % rate of identifying a true positive signal (in %)

Component_10pct_vssigs = N_10pct/length(Returns)*100;

N_5pct = sum(abs(Returns) > R_top5pct);

Disc_rate_5pct_vshist = N_5pct/(floor(length(RvsT_hist)*0.05))*100;

Component_5pct_vssigs = N_5pct/length(Returns)*100;

Disc_rate_10pct_vshist

Disc_rate_5pct_vshist

Component_10pct_vssigs

Component_5pct_vssigs

**Text I. Matlab script for reliability analysis (Figs 12 - 14)**

load('AUDJPY_standard_15s_90s.mat') % newM

load('V8_GF_AUDJPY_AR_15s_90s_gauswid100_Rwin225_Rst75_win80_inc1_ininc1_samp1000_band80_Phist0pt08.mat')

TI = 15; % time interval of data (s);

TW = 0.1; % time window for computing Returns

topxpct = 5; % the percentage of most extreme return%s to be classified as CT

Duration_EWS = 0.9; % the lasting duration (in days, weekends ruled out) of every qualified EWS

% (for every moment within it, a large return within future TW days is expected)

P_kendall_max = 0.05; % max P for kendall allowed for qualified EWS

P_endpoint_max = 0.04; % max P for endpoint allowed for qualified EWS

N_samples = 100000;

R_step = 75;

Size_sample = floor((24*3600/TI/R_step)*250); % 24*3600/TI/R_step for 1 day

len_sig = size(signal,1);

for i = 1:len_sig

if signal(i, 1) == 0

signal(i:len_sig, :) = []; % remove zeros

break

end

end

signal(isnan(signal(:,3)), :) = []; % remove NaNs if any

T_ind = (signal(1, 4):R_step:signal(end, 4))'; % start from the moment for the first EWS

T_ind(end) = []; % remove the very last one to avoid bugs

infoM = zeros(length(T_ind), 8); % the information matrix

infoM(:,1) = T_ind; % col1: time (index in newM)

infoM(:,2) = newM(T_ind, 1); % col2: time (real)

EWS_ind = 1; % index (in signal) of current/last EWS

EWS_now = signal(EWS_ind, :);

EWS_next = signal(EWS_ind+1, :);

DT5 = floor(5*24*3600/TI);

% first remove the tail in infoM where Return is too long to calculate

for j = 1:1000

if infoM(end+1-j, 1)+DT5 <= length(newM)

infoM(end+1-j:end, :) = [];

break

end

end

for i = 1:size(infoM, 1)

% check EWS records

if infoM(i, 1) >= EWS_next(4)

EWS_ind = EWS_ind+1;

EWS_now = signal(EWS_ind, :);

EWS_next = signal(EWS_ind+1, :);

end

infoM(i, 3) = infoM(i, 1) - EWS_now(4);

infoM(i, 4) = infoM(i, 2) - EWS_now(2);

infoM(i, 5:8) = EWS_now(5:8);

end

len_M = size(infoM, 1);

ReturnM = zeros(len_M, 4); % Return Matrix

ReturnM(:, 1:2) = infoM(:, 1:2); % col1, 2: time (index in newM), time (real)

% col3, 4: short-term future return percentage with time windows TW (days),

% and time (days) of occurrence (weekends ruled out)

DTmax = floor(TW*24*3600/TI); % window for calculation of Return (index in newM)

for i = 1:len_M

Price = newM(infoM(i, 1):(infoM(i, 1)+DTmax), :);

Price_diff = Price;

Price_diff(:,2) = Price(:,2) - Price(1,2);

[MaxP, MaxI] = max(abs(Price_diff(:,2))); % the maximum of abs(Price) and the index

ReturnM(i, 3) = 100*(Price(MaxI, 2) - Price(1, 2))/Price(1, 2); % Return %

ReturnM(i, 4) = MaxI*TI/3600/24; % time of occurrence

end

ReturnM(:, 3) = abs(ReturnM(:, 3)); % change to absolute value of return %

abs_sort_R = sort(ReturnM(:, 3), 'descend');

R_topxpct = abs_sort_R(floor(len_M*topxpct*0.01)); % windows with returns below which are not counted as CT

T_Duration_EWS = Duration_EWS*24*3600/TI; % Duration in indexes, to compare with infoM(:,3)

RESM = zeros(len_M, 2); % Return-EWS State Matrix, col1 for return, col2 for EWS

RESM(:,1) = (ReturnM(:,3)>R_topxpct)';

RESM(:,2) = ((infoM(:,3)<T_Duration_EWS)&(infoM(:,6)<=P_kendall_max)&(infoM(:,8)<=P_endpoint_max))';

P1 = sum(RESM(:,1)&RESM(:,2))/sum(RESM(:,2)); % P(Large Return|EWS) a.k.a. precision

P2 = sum(RESM(:,1)&(1-RESM(:,2)))/sum(1-RESM(:,2)); % P(Large Return|no EWS)

Ratio = P1/P2;

Ratios = zeros(N_samples, 1);

Precisions = zeros(N_samples, 1);

inds_start = randi(size(RESM,1)-Size_sample-1, 1, N_samples);

for i = 1:N_samples

ind_start = inds_start(i);

RESM_sample = RESM(ind_start:(ind_start+Size_sample), :);

P1_sample = sum(RESM_sample(:,1)&RESM_sample(:,2))/sum(RESM_sample(:,2)); % P(Large Return|EWS)

P2_sample = sum(RESM(:,1)&(1-RESM(:,2)))/sum(1-RESM(:,2)); % P(Large Return|no EWS)

Ratios(i) = P1_sample/P2_sample;

Precisions(i) = P1_sample;

end

N_Ratio_abv1 = sum(Ratios>1);

Rate_Ratio_abv1 = N_Ratio_abv1/N_samples;

%-------------------------------------------------------------------------------------------------------------

ax1 = subplot(3,2,1);

histogram(Ratios)

p1 = line([1, 1], get(ax1, 'YLim'), 'linewidth', 3, 'Color', 'r');

p2 = line([Ratio, Ratio], get(ax1, 'YLim'), 'linewidth', 3, 'Color', 'k');

legend([p1, p2], {['Rate of exceeding 1: ' num2str(Rate_Ratio_abv1)], ['Pool: ' num2str(Ratio)]}, 'FontSize', 30)

xlabel('AC(1) Sample P1/P2 Ratio', 'FontSize', 36)

ylabel({'(a)';'Count'}, 'FontSize', 36)

set(gca,'FontSize', 32, 'linewidth' ,2.5)

ax2 = subplot(3,2,2);

histogram(Precisions)

p3 = line([P1, P1], get(ax2, 'YLim'), 'linewidth', 3, 'Color', 'k');

legend(p3, {['Pool: ' num2str(P1)]}, 'FontSize', 30)

xlabel('AC(1) Sample Precision (P1)', 'FontSize', 36)

ylabel({'(b)';'Count'}, 'FontSize', 36)

set(gca,'FontSize', 32, 'linewidth' ,2.5)

%-------------------------------------------------------------------------------------------------------------

load('V8_GF_AUDJPY_Var_15s_90s_gauswid100_Rwin225_Rst75_win80_inc1_ininc1_samp1000_band80_Phist0pt08.mat')

len_sig = size(signal,1);

for i = 1:len_sig

if signal(i, 1) == 0

signal(i:len_sig, :) = []; % remove zeros

break

end

end

signal(isnan(signal(:,3)), :) = []; % remove NaNs if any

T_ind = (signal(1, 4):R_step:signal(end, 4))'; % start from the moment for the first EWS

T_ind(end) = []; % remove the very last one to avoid bugs

infoM = zeros(length(T_ind), 8); % the information matrix

infoM(:,1) = T_ind; % col1: time (index in newM)

infoM(:,2) = newM(T_ind, 1); % col2: time (real)

EWS_ind = 1; % index (in signal) of current/last EWS

EWS_now = signal(EWS_ind, :);

EWS_next = signal(EWS_ind+1, :);

DT5 = floor(5*24*3600/TI);

% first remove the tail in infoM where Return is too long to calculate

for j = 1:1000

if infoM(end+1-j, 1)+DT5 <= length(newM)

infoM(end+1-j:end, :) = [];

break

end

end

for i = 1:size(infoM, 1)

% check EWS records

if infoM(i, 1) >= EWS_next(4)

EWS_ind = EWS_ind+1;

EWS_now = signal(EWS_ind, :);

EWS_next = signal(EWS_ind+1, :);

end

infoM(i, 3) = infoM(i, 1) - EWS_now(4);

infoM(i, 4) = infoM(i, 2) - EWS_now(2);

infoM(i, 5:8) = EWS_now(5:8);

end

len_M = size(infoM, 1);

ReturnM = zeros(len_M, 4); % Return Matrix

ReturnM(:, 1:2) = infoM(:, 1:2); % col1, 2: time (index in newM), time (real)

% col3, 4: short-term future return percentage with time windows TW (days),

% and time (days) of occurrence (weekends ruled out)

DTmax = floor(TW*24*3600/TI); % window for calculation of Return (index in newM)

for i = 1:len_M

Price = newM(infoM(i, 1):(infoM(i, 1)+DTmax), :);

Price_diff = Price;

Price_diff(:,2) = Price(:,2) - Price(1,2);

[MaxP, MaxI] = max(abs(Price_diff(:,2))); % the maximum of abs(Price) and the index

ReturnM(i, 3) = 100*(Price(MaxI, 2) - Price(1, 2))/Price(1, 2); % Return %

ReturnM(i, 4) = MaxI*TI/3600/24; % time of occurrence

end

ReturnM(:, 3) = abs(ReturnM(:, 3)); % change to absolute value of return %

abs_sort_R = sort(ReturnM(:, 3), 'descend');

R_topxpct = abs_sort_R(floor(len_M*topxpct*0.01)); % windows with returns below which are not counted as CT

T_Duration_EWS = Duration_EWS*24*3600/TI; % Duration in indexes, to compare with infoM(:,3)

RESM = zeros(len_M, 2); % Return-EWS State Matrix, col1 for return, col2 for EWS

RESM(:,1) = (ReturnM(:,3)>R_topxpct)';

RESM(:,2) = ((infoM(:,3)<T_Duration_EWS)&(infoM(:,6)<=P_kendall_max)&(infoM(:,8)<=P_endpoint_max))';

P1 = sum(RESM(:,1)&RESM(:,2))/sum(RESM(:,2)); % P(Large Return|EWS) a.k.a. precision

P2 = sum(RESM(:,1)&(1-RESM(:,2)))/sum(1-RESM(:,2)); % P(Large Return|no EWS)

Ratio = P1/P2;

Ratios = zeros(N_samples, 1);

Precisions = zeros(N_samples, 1);

inds_start = randi(size(RESM,1)-Size_sample-1, 1, N_samples);

for i = 1:N_samples

ind_start = inds_start(i);

RESM_sample = RESM(ind_start:(ind_start+Size_sample), :);

P1_sample = sum(RESM_sample(:,1)&RESM_sample(:,2))/sum(RESM_sample(:,2)); % P(Large Return|EWS)

P2_sample = sum(RESM(:,1)&(1-RESM(:,2)))/sum(1-RESM(:,2)); % P(Large Return|no EWS)

Ratios(i) = P1_sample/P2_sample;

Precisions(i) = P1_sample;

end

N_Ratio_abv1 = sum(Ratios>1);

Rate_Ratio_abv1 = N_Ratio_abv1/N_samples;

%-------------------------------------------------------------------------------------------------------------

ax3 = subplot(3,2,3);

histogram(Ratios)

p1 = line([1, 1], get(ax1, 'YLim'), 'linewidth', 3, 'Color', 'r');

p2 = line([Ratio, Ratio], get(ax1, 'YLim'), 'linewidth', 3, 'Color', 'k');

legend([p1, p2], {['Rate of exceeding 1: ' num2str(Rate_Ratio_abv1)], ['Pool: ' num2str(Ratio)]}, 'FontSize', 30)

xlabel('Var Sample P1/P2 Ratio', 'FontSize', 36)

ylabel({'(c)';'Count'}, 'FontSize', 36)

set(gca,'FontSize', 32, 'linewidth' ,2.5)

ax4 = subplot(3,2,4);

histogram(Precisions)

p3 = line([P1, P1], get(ax2, 'YLim'), 'linewidth', 3, 'Color', 'k');

legend(p3, {['Pool: ' num2str(P1)]}, 'FontSize', 30)

xlabel('Var Sample Precision (P1)', 'FontSize', 36)

ylabel({'(d)';'Count'}, 'FontSize', 36)

set(gca,'FontSize', 32, 'linewidth' ,2.5)

%-------------------------------------------------------------------------------------------------------------

load('V8_GF_AUDJPY_LFPS_Crit26_15s_90s_gauswid100_Rwin225_Rst75_win80_inc1_ininc1_samp1000_band80_Phist0pt08.mat')

len_sig = size(signal,1);

for i = 1:len_sig

if signal(i, 1) == 0

signal(i:len_sig, :) = []; % remove zeros

break

end

end

signal(isnan(signal(:,3)), :) = []; % remove NaNs if any

T_ind = (signal(1, 4):R_step:signal(end, 4))'; % start from the moment for the first EWS

T_ind(end) = []; % remove the very last one to avoid bugs

infoM = zeros(length(T_ind), 8); % the information matrix

infoM(:,1) = T_ind; % col1: time (index in newM)

infoM(:,2) = newM(T_ind, 1); % col2: time (real)

EWS_ind = 1; % index (in signal) of current/last EWS

EWS_now = signal(EWS_ind, :);

EWS_next = signal(EWS_ind+1, :);

DT5 = floor(5*24*3600/TI);

% first remove the tail in infoM where Return is too long to calculate

for j = 1:1000

if infoM(end+1-j, 1)+DT5 <= length(newM)

infoM(end+1-j:end, :) = [];

break

end

end

for i = 1:size(infoM, 1)

% check EWS records

if infoM(i, 1) >= EWS_next(4)

EWS_ind = EWS_ind+1;

EWS_now = signal(EWS_ind, :);

EWS_next = signal(EWS_ind+1, :);

end

infoM(i, 3) = infoM(i, 1) - EWS_now(4);

infoM(i, 4) = infoM(i, 2) - EWS_now(2);

infoM(i, 5:8) = EWS_now(5:8);

end

len_M = size(infoM, 1);

ReturnM = zeros(len_M, 4); % Return Matrix

ReturnM(:, 1:2) = infoM(:, 1:2); % col1, 2: time (index in newM), time (real)

% col3, 4: short-term future return percentage with time windows TW (days),

% and time (days) of occurrence (weekends ruled out)

DTmax = floor(TW*24*3600/TI); % window for calculation of Return (index in newM)

for i = 1:len_M

Price = newM(infoM(i, 1):(infoM(i, 1)+DTmax), :);

Price_diff = Price;

Price_diff(:,2) = Price(:,2) - Price(1,2);

[MaxP, MaxI] = max(abs(Price_diff(:,2))); % the maximum of abs(Price) and the index

ReturnM(i, 3) = 100*(Price(MaxI, 2) - Price(1, 2))/Price(1, 2); % Return %

ReturnM(i, 4) = MaxI*TI/3600/24; % time of occurrence

end

ReturnM(:, 3) = abs(ReturnM(:, 3)); % change to absolute value of return %

abs_sort_R = sort(ReturnM(:, 3), 'descend');

R_topxpct = abs_sort_R(floor(len_M*topxpct*0.01)); % windows with returns below which are not counted as CT

T_Duration_EWS = Duration_EWS*24*3600/TI; % Duration in indexes, to compare with infoM(:,3)

RESM = zeros(len_M, 2); % Return-EWS State Matrix, col1 for return, col2 for EWS

RESM(:,1) = (ReturnM(:,3)>R_topxpct)';

RESM(:,2) = ((infoM(:,3)<T_Duration_EWS)&(infoM(:,6)<=P_kendall_max)&(infoM(:,8)<=P_endpoint_max))';

P1 = sum(RESM(:,1)&RESM(:,2))/sum(RESM(:,2)); % P(Large Return|EWS) a.k.a. precision

P2 = sum(RESM(:,1)&(1-RESM(:,2)))/sum(1-RESM(:,2)); % P(Large Return|no EWS)

Ratio = P1/P2;

Ratios = zeros(N_samples, 1);

Precisions = zeros(N_samples, 1);

inds_start = randi(size(RESM,1)-Size_sample-1, 1, N_samples);

for i = 1:N_samples

ind_start = inds_start(i);

RESM_sample = RESM(ind_start:(ind_start+Size_sample), :);

P1_sample = sum(RESM_sample(:,1)&RESM_sample(:,2))/sum(RESM_sample(:,2)); % P(Large Return|EWS)

P2_sample = sum(RESM(:,1)&(1-RESM(:,2)))/sum(1-RESM(:,2)); % P(Large Return|no EWS)

Ratios(i) = P1_sample/P2_sample;

Precisions(i) = P1_sample;

end

N_Ratio_abv1 = sum(Ratios>1);

Rate_Ratio_abv1 = N_Ratio_abv1/N_samples;

%-------------------------------------------------------------------------------------------------------------

ax5 = subplot(3,2,5);

histogram(Ratios)

p1 = line([1, 1], get(ax1, 'YLim'), 'linewidth', 3, 'Color', 'r');

p2 = line([Ratio, Ratio], get(ax1, 'YLim'), 'linewidth', 3, 'Color', 'k');

legend([p1, p2], {['Rate of exceeding 1: ' num2str(Rate_Ratio_abv1)], ['Pool: ' num2str(Ratio)]}, 'FontSize', 30)

xlabel('LFPS Sample P1/P2 Ratio', 'FontSize', 36)

ylabel({'(e)';'Count'}, 'FontSize', 36)

set(gca,'FontSize', 32, 'linewidth' ,2.5)

ax6 = subplot(3,2,6);

histogram(Precisions)

p3 = line([P1, P1], get(ax2, 'YLim'), 'linewidth', 3, 'Color', 'k');

legend(p3, {['Pool: ' num2str(P1)]}, 'FontSize', 30)

xlabel('LFPS Sample Precision (P1)', 'FontSize', 36)

ylabel({'(f)';'Count'}, 'FontSize', 36)

set(gca,'FontSize', 32, 'linewidth' ,2.5)

**Text J. Matlab script for reliability analysis (Fig 15)**

tic

load('AUDJPY_standard_15s_90s.mat') % newM

load('V8_GF_AUDJPY_AR_15s_90s_gauswid100_Rwin225_Rst75_win80_inc1_ininc1_samp1000_band80_Phist0pt08.mat')

TI = 15; % time interval of data (s);

TW = 0.1; % time window for computing Returns

topxpct = 5; % the percentage of most extreme return%s to be classified as CT

Duration_EWS = 0.9; % the lasting duration (in days, weekends ruled out) of every qualified EWS

% (for every moment within it, a large return within future TW days is expected)

P_kendall_max = 0.05; % max P for kendall allowed for qualified EWS

P_endpoint_max = 0.04; % max P for endpoint allowed for qualified EWS

N_samples = 100000;

R_step = 75;

Size_Sample_days = 10:10:260;

Size_Sample = floor((24*3600/TI/R_step)*Size_Sample_days); % 24*3600/TI/R_step for 1 day

len_sig = size(signal,1);

for i = 1:len_sig

if signal(i, 1) == 0

signal(i:len_sig, :) = []; % remove zeros

break

end

end

signal(isnan(signal(:,3)), :) = []; % remove NaNs if any

T_ind = (signal(1, 4):R_step:signal(end, 4))'; % start from the moment for the first EWS

T_ind(end) = []; % remove the very last one to avoid bugs

infoM = zeros(length(T_ind), 8); % the information matrix

infoM(:,1) = T_ind; % col1: time (index in newM)

infoM(:,2) = newM(T_ind, 1); % col2: time (real)

EWS_ind = 1; % index (in signal) of current/last EWS

EWS_now = signal(EWS_ind, :);

EWS_next = signal(EWS_ind+1, :);

DT5 = floor(5*24*3600/TI);

% first remove the tail in infoM where Return is too long to calculate

for j = 1:1000

if infoM(end+1-j, 1)+DT5 <= length(newM)

infoM(end+1-j:end, :) = [];

break

end

end

for i = 1:size(infoM, 1)

% check EWS records

if infoM(i, 1) >= EWS_next(4)

EWS_ind = EWS_ind+1;

EWS_now = signal(EWS_ind, :);

EWS_next = signal(EWS_ind+1, :);

end

infoM(i, 3) = infoM(i, 1) - EWS_now(4);

infoM(i, 4) = infoM(i, 2) - EWS_now(2);

infoM(i, 5:8) = EWS_now(5:8);

end

len_M = size(infoM, 1);

ReturnM = zeros(len_M, 4); % Return Matrix

ReturnM(:, 1:2) = infoM(:, 1:2); % col1, 2: time (index in newM), time (real)

% col3, 4: short-term future return percentage with time windows TW (days),

% and time (days) of occurrence (weekends ruled out)

DTmax = floor(TW*24*3600/TI); % window for calculation of Return (index in newM)

for i = 1:len_M

Price = newM(infoM(i, 1):(infoM(i, 1)+DTmax), :);

Price_diff = Price;

Price_diff(:,2) = Price(:,2) - Price(1,2);

[MaxP, MaxI] = max(abs(Price_diff(:,2))); % the maximum of abs(Price) and the index

ReturnM(i, 3) = 100*(Price(MaxI, 2) - Price(1, 2))/Price(1, 2); % Return %

ReturnM(i, 4) = MaxI*TI/3600/24; % time of occurrence

end

ReturnM(:, 3) = abs(ReturnM(:, 3)); % change to absolute value of return %

abs_sort_R = sort(ReturnM(:, 3), 'descend');

R_topxpct = abs_sort_R(floor(len_M*topxpct*0.01)); % windows with returns below which are not counted as CT

T_Duration_EWS = Duration_EWS*24*3600/TI; % Duration in indexes, to compare with infoM(:,3)

RESM = zeros(len_M, 2); % Return-EWS State Matrix, col1 for return, col2 for EWS

RESM(:,1) = (ReturnM(:,3)>R_topxpct)';

RESM(:,2) = ((infoM(:,3)<T_Duration_EWS)&(infoM(:,6)<=P_kendall_max)&(infoM(:,8)<=P_endpoint_max))';

%rate_EWS = sum(RESM(:,2))/len_M; % rate of total large return (rate of large return is topxpct%)

P1_AR = sum(RESM(:,1)&RESM(:,2))/sum(RESM(:,2)); % P(Large Return|EWS) a.k.a. precision

P2_AR = sum(RESM(:,1)&(1-RESM(:,2)))/sum(1-RESM(:,2)); % P(Large Return|no EWS)

Ratio_AR = P1_AR/P2_AR;

Ratios_low_5pct_AR = zeros(length(Size_Sample), 1);

Precisions_low_5pct_AR = zeros(length(Size_Sample), 1);

inds = 1:size(RESM, 1);

for j = 1:length(Size_Sample)

Size_sample = Size_Sample(j);

Ratios = zeros(N_samples, 1);

Precisions = zeros(N_samples, 1);

for i = 1:N_samples

inds_chosen = datasample(inds, Size_sample)';

RESM_sample = RESM(inds_chosen, :);

P1_sample = sum(RESM_sample(:,1)&RESM_sample(:,2))/sum(RESM_sample(:,2)); % P(Large Return|EWS)

P2_sample = sum(RESM(:,1)&(1-RESM(:,2)))/sum(1-RESM(:,2)); % P(Large Return|no EWS)

Ratios(i) = P1_sample/P2_sample;

Precisions(i) = P1_sample;

end

S_Ratios = sort(Ratios);

Ratios_low_5pct_AR(j) = S_Ratios(floor(N_samples*0.05));

S_Precisions = sort(Precisions);

Precisions_low_5pct_AR(j) = S_Precisions(floor(N_samples*0.05));

end

%-------------------------------------------------------------------------------------------------------------

load('V8_GF_AUDJPY_Var_15s_90s_gauswid100_Rwin225_Rst75_win80_inc1_ininc1_samp1000_band80_Phist0pt08.mat')

len_sig = size(signal,1);

for i = 1:len_sig

if signal(i, 1) == 0

signal(i:len_sig, :) = []; % remove zeros

break

end

end

signal(isnan(signal(:,3)), :) = []; % remove NaNs if any

T_ind = (signal(1, 4):R_step:signal(end, 4))'; % start from the moment for the first EWS

T_ind(end) = []; % remove the very last one to avoid bugs

infoM = zeros(length(T_ind), 8); % the information matrix

infoM(:,1) = T_ind; % col1: time (index in newM)

infoM(:,2) = newM(T_ind, 1); % col2: time (real)

EWS_ind = 1; % index (in signal) of current/last EWS

EWS_now = signal(EWS_ind, :);

EWS_next = signal(EWS_ind+1, :);

DT5 = floor(5*24*3600/TI);

% first remove the tail in infoM where Return is too long to calculate

for j = 1:1000

if infoM(end+1-j, 1)+DT5 <= length(newM)

infoM(end+1-j:end, :) = [];

break

end

end

for i = 1:size(infoM, 1)

% check EWS records

if infoM(i, 1) >= EWS_next(4)

EWS_ind = EWS_ind+1;

EWS_now = signal(EWS_ind, :);

EWS_next = signal(EWS_ind+1, :);

end

infoM(i, 3) = infoM(i, 1) - EWS_now(4);

infoM(i, 4) = infoM(i, 2) - EWS_now(2);

infoM(i, 5:8) = EWS_now(5:8);

end

len_M = size(infoM, 1);

ReturnM = zeros(len_M, 4); % Return Matrix

ReturnM(:, 1:2) = infoM(:, 1:2); % col1, 2: time (index in newM), time (real)

% col3, 4: short-term future return percentage with time windows TW (days),

% and time (days) of occurrence (weekends ruled out)

DTmax = floor(TW*24*3600/TI); % window for calculation of Return (index in newM)

for i = 1:len_M

Price = newM(infoM(i, 1):(infoM(i, 1)+DTmax), :);

Price_diff = Price;

Price_diff(:,2) = Price(:,2) - Price(1,2);

[MaxP, MaxI] = max(abs(Price_diff(:,2))); % the maximum of abs(Price) and the index

ReturnM(i, 3) = 100*(Price(MaxI, 2) - Price(1, 2))/Price(1, 2); % Return %

ReturnM(i, 4) = MaxI*TI/3600/24; % time of occurrence

end

ReturnM(:, 3) = abs(ReturnM(:, 3)); % change to absolute value of return %

abs_sort_R = sort(ReturnM(:, 3), 'descend');

R_topxpct = abs_sort_R(floor(len_M*topxpct*0.01)); % windows with returns below which are not counted as CT

T_Duration_EWS = Duration_EWS*24*3600/TI; % Duration in indexes, to compare with infoM(:,3)

RESM = zeros(len_M, 2); % Return-EWS State Matrix, col1 for return, col2 for EWS

RESM(:,1) = (ReturnM(:,3)>R_topxpct)';

RESM(:,2) = ((infoM(:,3)<T_Duration_EWS)&(infoM(:,6)<=P_kendall_max)&(infoM(:,8)<=P_endpoint_max))';

%rate_EWS = sum(RESM(:,2))/len_M; % rate of total large return (rate of large return is topxpct%)

P1_Var = sum(RESM(:,1)&RESM(:,2))/sum(RESM(:,2)); % P(Large Return|EWS) a.k.a. precision

P2_Var = sum(RESM(:,1)&(1-RESM(:,2)))/sum(1-RESM(:,2)); % P(Large Return|no EWS)

Ratio_Var = P1_Var/P2_Var;

Ratios_low_5pct_Var = zeros(length(Size_Sample), 1);

Precisions_low_5pct_Var = zeros(length(Size_Sample), 1);

inds = 1:size(RESM, 1);

for j = 1:length(Size_Sample)

Size_sample = Size_Sample(j);

Ratios = zeros(N_samples, 1);

Precisions = zeros(N_samples, 1);

for i = 1:N_samples

inds_chosen = datasample(inds, Size_sample)';

RESM_sample = RESM(inds_chosen, :);

P1_sample = sum(RESM_sample(:,1)&RESM_sample(:,2))/sum(RESM_sample(:,2)); % P(Large Return|EWS)

P2_sample = sum(RESM(:,1)&(1-RESM(:,2)))/sum(1-RESM(:,2)); % P(Large Return|no EWS)

Ratios(i) = P1_sample/P2_sample;

Precisions(i) = P1_sample;

end

S_Ratios = sort(Ratios);

Ratios_low_5pct_Var(j) = S_Ratios(floor(N_samples*0.05));

S_Precisions = sort(Precisions);

Precisions_low_5pct_Var(j) = S_Precisions(floor(N_samples*0.05));

end

%-------------------------------------------------------------------------------------------------------------

load('V8_GF_AUDJPY_LFPS_Crit26_15s_90s_gauswid100_Rwin225_Rst75_win80_inc1_ininc1_samp1000_band80_Phist0pt08.mat')

len_sig = size(signal,1);

for i = 1:len_sig

if signal(i, 1) == 0

signal(i:len_sig, :) = []; % remove zeros

break

end

end

signal(isnan(signal(:,3)), :) = []; % remove NaNs if any

T_ind = (signal(1, 4):R_step:signal(end, 4))'; % start from the moment for the first EWS

T_ind(end) = []; % remove the very last one to avoid bugs

infoM = zeros(length(T_ind), 8); % the information matrix

infoM(:,1) = T_ind; % col1: time (index in newM)

infoM(:,2) = newM(T_ind, 1); % col2: time (real)

EWS_ind = 1; % index (in signal) of current/last EWS

EWS_now = signal(EWS_ind, :);

EWS_next = signal(EWS_ind+1, :);

DT5 = floor(5*24*3600/TI);

% first remove the tail in infoM where Return is too long to calculate

for j = 1:1000

if infoM(end+1-j, 1)+DT5 <= length(newM)

infoM(end+1-j:end, :) = [];

break

end

end

for i = 1:size(infoM, 1)

% check EWS records

if infoM(i, 1) >= EWS_next(4)

EWS_ind = EWS_ind+1;

EWS_now = signal(EWS_ind, :);

EWS_next = signal(EWS_ind+1, :);

end

infoM(i, 3) = infoM(i, 1) - EWS_now(4);

infoM(i, 4) = infoM(i, 2) - EWS_now(2);

infoM(i, 5:8) = EWS_now(5:8);

end

len_M = size(infoM, 1);

ReturnM = zeros(len_M, 4); % Return Matrix

ReturnM(:, 1:2) = infoM(:, 1:2); % col1, 2: time (index in newM), time (real)

% col3, 4: short-term future return percentage with time windows TW (days),

% and time (days) of occurrence (weekends ruled out)

DTmax = floor(TW*24*3600/TI); % window for calculation of Return (index in newM)

for i = 1:len_M

Price = newM(infoM(i, 1):(infoM(i, 1)+DTmax), :);

Price_diff = Price;

Price_diff(:,2) = Price(:,2) - Price(1,2);

[MaxP, MaxI] = max(abs(Price_diff(:,2))); % the maximum of abs(Price) and the index

ReturnM(i, 3) = 100*(Price(MaxI, 2) - Price(1, 2))/Price(1, 2); % Return %

ReturnM(i, 4) = MaxI*TI/3600/24; % time of occurrence

end

ReturnM(:, 3) = abs(ReturnM(:, 3)); % change to absolute value of return %

abs_sort_R = sort(ReturnM(:, 3), 'descend');

R_topxpct = abs_sort_R(floor(len_M*topxpct*0.01)); % windows with returns below which are not counted as CT

T_Duration_EWS = Duration_EWS*24*3600/TI; % Duration in indexes, to compare with infoM(:,3)

RESM = zeros(len_M, 2); % Return-EWS State Matrix, col1 for return, col2 for EWS

RESM(:,1) = (ReturnM(:,3)>R_topxpct)';

RESM(:,2) = ((infoM(:,3)<T_Duration_EWS)&(infoM(:,6)<=P_kendall_max)&(infoM(:,8)<=P_endpoint_max))';

%rate_EWS = sum(RESM(:,2))/len_M; % rate of total large return (rate of large return is topxpct%)

P1_LFPS = sum(RESM(:,1)&RESM(:,2))/sum(RESM(:,2)); % P(Large Return|EWS) a.k.a. precision

P2_LFPS = sum(RESM(:,1)&(1-RESM(:,2)))/sum(1-RESM(:,2)); % P(Large Return|no EWS)

Ratio_LFPS = P1_LFPS/P2_LFPS;

Ratios_low_5pct_LFPS = zeros(length(Size_Sample), 1);

Precisions_low_5pct_LFPS = zeros(length(Size_Sample), 1);

inds = 1:size(RESM, 1);

for j = 1:length(Size_Sample)

Size_sample = Size_Sample(j);

Ratios = zeros(N_samples, 1);

Precisions = zeros(N_samples, 1);

for i = 1:N_samples

inds_chosen = datasample(inds, Size_sample)';

RESM_sample = RESM(inds_chosen, :);

P1_sample = sum(RESM_sample(:,1)&RESM_sample(:,2))/sum(RESM_sample(:,2)); % P(Large Return|EWS)

P2_sample = sum(RESM(:,1)&(1-RESM(:,2)))/sum(1-RESM(:,2)); % P(Large Return|no EWS)

Ratios(i) = P1_sample/P2_sample;

Precisions(i) = P1_sample;

end

S_Ratios = sort(Ratios);

Ratios_low_5pct_LFPS(j) = S_Ratios(floor(N_samples*0.05));

S_Precisions = sort(Precisions);

Precisions_low_5pct_LFPS(j) = S_Precisions(floor(N_samples*0.05));

end

save('conditinoal_prob_test_AUDJPY_variableS_results.mat')

toc
