# Supplementary material for: How one might miss early warning signals of critical transitions in time series data: A systematic study of two major currency pairs
Source: PLoS One. 2018 Mar 14;13(3):e0191439. doi: 10.1371/journal.pone.0191439 (PMC5851542; doi:10.1371/journal.pone.0191439)
Supplement: S1 Appendix — (DOCX) [file pone.0191439.s002.docx]

How One Might Miss Early Warning Signals of Critical Transitions in Time Series Data: A Systematic Study of Two Major Currency Pairs

S1 Support Information: Tables in Sensitivity Analyses

Haoyu Wen,^1,2*^ Massimo Pica Ciamarra,^1,2^  Siew Ann Cheong^1,2^

^1^ Division of Physics and Applied Physics, School of Physical and Mathematical Sciences, Nanyang Technological University, Singapore

^2^ Complexity Institute, Nanyang Technological University, Singapore

* Corresponding author

Email: [s160049@e.ntu.edu.sg](mailto:s160049@e.ntu.edu.sg) (H. W)

**List of contents**

- Table A. Sensitivity analysis of the optimal discovery rates and specificities of statistically significant EWSs from AC(1), Var, and LFPS, for the AUD-JPY exchange rate from 1996 to 2004. Text A. Matlab script for converting original data into fixed time intervals (15s or 30s)
- Table B. Sensitivity analysis of the optimal discovery rates and specificities of statistically significant EWSs for the AUD-JPY exchange rate from 2005 to 2010.
- Table C. Sensitivity analysis of the optimal discovery rates and specificities of statistically significant EWSs for the CHF-JPY exchange rates from 2008 to 2009.
- Table D. Average percentage changes of DR5 and SP5 for 1% changes of the parameters around the optimal values, of EWSs from AC(1), Var, and LFPS, for all three data sets. Derived from Tables A, B, and C.

**Table A.** **Sensitivity analysis of the optimal discovery rates and specificities of statistically significant EWSs from AC(1), Var, and LFPS, for the AUD-JPY exchange rate from 1996 to 2004.**

| $T_{0}$ | $R_{win}$ | $R_{ind}$ | $\sigma$ | $P$ | ${DR}_{10}$ (%) | ${DR}_{5}$ (%) | ${SP}_{10}$ (%) | ${SP}_{5}$ (%) |
| --- | --- | --- | --- | --- | --- | --- | --- | --- |
| 30 | 120 | 80 | 76 | 10 | 5.35, 4.49, 5.42 | 5.59, 4.76, 5.79 | 17.42, 14.74, 17.87 | 9.11, 7.81, 9.54 |
| 45 | 80 | 80 | 50 | 10 | 4.95, 5.67, 4.87 | 6.10, 6.41, 5.60 | 18.02, 19.19, 18.13 | 11.10, 10.86, 10.40 |
| 60 | 60 | 80 | 38 | 10 | 4.54, 5.94, 4.26 | 5.56, 7.02, 5.29 | 17.50, 19.02, 17.85 | 10.73, 11.23, 11.07 |
| 30 | 120 | 80 | 76 | 10 | 5.35, 4.49, 5.42 | 5.59, 4.76, 5.79 | 17.42, 14.74, 17.87 | 9.11, 7.81, 9.54 |
| 30 | 120 | 80 | 70 | 10 | 5.26, 4.45, 5.38 | 5.66, 4.72, 5.68 | 17.29, 14.74, 17.86 | 9.35, 7.79, 9.43 |
| 30 | 120 | 80 | 82 | 10 | 5.33, 4.51, 5.40 | 5.66, 4.73, 5.85 | 17.41, 14.79, 17.81 | 9.25, 7.76, 9.65 |
| 30 | 120 | 80 | 76 | 10 | 5.35, 4.49, 5.42 | 5.59, 4.76, 5.79 | 17.42, 14.74, 17.87 | 9.11, 7.81, 9.54 |
| 30 | 120 | 86 | 76 | 10 | 5.44, 4.36, 5.34 | 5.72, 4.29, 5.44 | 17.58, 14.57, 17.36 | 9.24, 7.16, 8.83 |
| 30 | 120 | 74 | 76 | 10 | 5.22, 4.68, 5.20 | 5.83, 5.29, 5.53 | 17.31, 15.01, 17.52 | 9.67, 8.48, 9.32 |
| 30 | 120 | 80 | 76 | 10 | 5.35, 4.49, 5.42 | 5.59, 4.76, 5.79 | 17.42, 14.74, 17.87 | 9.11, 7.81, 9.54 |
| 30 | 105 | 80 | 76 | 10 | 4.95, 4.80, 4.72 | 5.51, 5.38, 5.21 | 16.44, 14.90, 16.25 | 9.16, 8.25, 8.98 |
| 30 | 135 | 80 | 76 | 10 | 5.91, 4.28, 5.81 | 6.41, 4.68, 6.05 | 18.89, 14.54, 18.47 | 10.25, 7.95, 9.62 |
| 30 | 150 | 80 | 76 | 10 | 6.10, 3.80, 5.93 | 6.78, 3.62, 6.40 | 18.89, 13.60, 18.64 | 10.50, 6.47, 10.06 |
| 30 | 135 | 80 | 76 | 10 | 5.91, 4.28, 5.81 | 6.41, 4.68, 6.05 | 18.89, 14.54, 18.47 | 10.25, 7.95, 9.62 |
| 30 | 147 | 74 | 76 | 10 | 5.67, 4.19, 5.50 | 5.91, 4.20, 5.57 | 18.42, 14.72, 18.36 | 9.59, 7.38, 9.29 |
| 30 | 126 | 86 | 76 | 10 | 6.07, 4.38, 5.86 | 6.55, 4.55, 6.14 | 18.91, 14.73, 18.86 | 10.21, 7.65, 9.88 |
| 30 | 114 | 92 | 76 | 10 | 5.75, 4.29, 5.70 | 6.33, 4.62, 5.95 | 18.18, 14.07, 18.17 | 10.01, 7.58, 9.47 |
| 30 | 126 | 86 | 76 | 10 | 5.86 | 6.14 | 18.86 | 9.88 |
| 30 | 126 | 86 | 76 | 8 | 5.85 | 6.19 | 18.76 | 9.92 |
| 30 | 126 | 86 | 76 | 12 | 5.64 | 6.01 | 18.39 | 9.80 |
| 30 | 126 | 86 | 76 | 6 | 6.27 | 7.27 | 19.74 | 11.45 |
| 30 | 126 | 86 | 76 | 3 | 3.60 | 3.65 | 14.88 | 7.53 |
| 30 | 126 | 86 | 76 | 4 | 4.92 | 5.99 | 17.23 | 10.49 |
| 30 | 126 | 86 | 76 | 5 | 5.65 | 6.62 | 17.87 | 10.47 |

The parameters were optimized sequentially, starting from $T_{0}$, and ending with $P$. We varied $T_{0}$ from 30 s to 45 s to 60 s, adjusting $R_{win}$ to keep $R_{ind}$ constant, and also adjusted$\sigma$ such that the product of $\sigma$ and $T_{0}$ is constant. This product is the time duration of the bandwidth for Gaussian smoothing. We then picked the value of $T_{0}$ that maximizes the discovery rate and specificities, and kept it fixed for the rest of the optimization. The next parameter we varied is $\sigma$, and as we varied it from 70 to 76 to 82, the optimal $\sigma$ was found to be 76. We then proceeded to vary $R_{ind}$, and thereafter $R_{win}$, and thence a combination of $R_{ind}$ and $R_{win}$ with their product kept roughly constant, before finally varying $P$. In this table, the parameter being varied is highlighted in green, while the optimal discovery rates and specificities are highlighted in yellow.

**Table B. Sensitivity analysis of the optimal discovery rates and specificities of statistically significant EWSs for the AUD-JPY exchange rate from 2005 to 2010.**

| $T_{0}$ | $R_{win}$ | $R_{ind}$ | $\sigma$ | $P$ | ${DR}_{10}$ (%) | ${DR}_{5}$ (%) | ${SP}_{10}$ (%) | ${SP}_{5}$ (%) |
| --- | --- | --- | --- | --- | --- | --- | --- | --- |
| 15 | 225 | 80 | 150 | 6 | 6.91, 10.13, 6.41 | 8.29, 13.15, 8.27 | 29.09, 42.33, 29.92 | 17.45, 27.49, 19.31 |
| 20 | 168 | 80 | 112 | 6 | 6.82, 10.30, 5.92 | 7.67, 13.38, 6.06 | 29.64, 42.58, 27.36 | 16.67, 27.66, 14.02 |
| 30 | 114 | 80 | 76 | 6 | 6.25, 9.75, 5.37 | 6.81, 12.88, 5.88 | 28.28, 41.23, 25.92 | 15.39, 27.25, 14.18 |
| 15 | 225 | 80 | 180 | 6 | 6.88, 10.02, 5.99 | 8.57, 13.27, 6.87 | 29.12, 43.69, 29.62 | 18.13, 28.94, 16.97 |
| 15 | 225 | 80 | 170 | 6 | 7.02, 10.06, 6.20 | 8.53, 13.15, 7.06 | 29.32, 43.33, 29.80 | 17.80, 28.33, 16.98 |
| 15 | 225 | 80 | 160 | 6 | 7.00, 10.12, 6.21 | 8.45, 13.17, 7.20 | 29.45, 42.91, 28.91 | 17.77, 27.93, 16.77 |
| 15 | 225 | 80 | 150 | 6 | 6.91, 10.13, 6.41 | 8.29, 13.15, 8.27 | 29.09, 42.33, 29.92 | 17.45, 27.49, 19.31 |
| 15 | 225 | 80 | 140 | 6 | 6.95, 10.15, 6.26 | 8.43, 13.15, 7.30 | 29.04, 42.20, 27.83 | 17.61, 27.35, 16.23 |
| 15 | 225 | 80 | 130 | 6 | 7.00, 10.36, 6.38 | 8.47, 13.39, 7.52 | 28.91, 42.42, 27.70 | 17.49, 27.43, 16.33 |
| 15 | 225 | 80 | 120 | 6 | 7.11, 10.44, 6.29 | 8.63, 13.73, 7.48 | 29.39, 42.16, 26.89 | 17.83, 27.74, 15.99 |
| 15 | 225 | 80 | 110 | 6 | 7.21, 10.39, 6.51 | 8.81, 13.63, 7.70 | 29.76, 41.42, 27.46 | 18.17, 27.18, 16.24 |
| 15 | 225 | 80 | 100 | 6 | 7.37, 10.27, 6.53 | 9.09, 13.61, 7.78 | 30.34, 40.78, 27.31 | 18.70, 27.03, 16.27 |
| 15 | 225 | 80 | 90 | 6 | 7.28, 10.26, 6.42 | 9.03, 13.69, 7.58 | 30.43, 40.60, 27.37 | 18.86, 27.09, 16.16 |
| 15 | 225 | 72 | 100 | 6 | 9.34, 11.42, 7.57 | 10.79, 15.38, 8.33 | 28.69, 38.73, 27.00 | 16.58, 26.08, 14.86 |
| 15 | 225 | 76 | 100 | 6 | 8.34, 10.97, 7.04 | 9.86, 14.66, 8.11 | 29.36, 39.63, 27.09 | 17.35, 26.48, 15.60 |
| 15 | 225 | 80 | 100 | 6 | 7.37, 10.27, 6.53 | 9.09, 13.61, 7.78 | 30.34, 40.78, 27.31 | 18.70, 27.03, 16.27 |
| 15 | 225 | 84 | 100 | 6 | 6.61, 9.76, 6.22 | 8.13, 12.82, 7.18 | 31.67, 41.45, 28.64 | 19.50, 27.21, 16.54 |
| 15 | 225 | 88 | 100 | 6 | 5.77, 9.82, 6.35 | 7.12, 13.41, 7.50 | 32.03, 43.21, 31.01 | 19.76, 29.51, 18.31 |
| 15 | 201 | 80 | 100 | 6 | 9.06, 11.72, 7.83 | 10.58, 15.74, 8.58 | 27.13, 38.58, 26.59 | 15.85, 25.91, 14.57 |
| 15 | 213 | 80 | 100 | 6 | 8.19, 11.09, 7.18 | 9.32, 14.91, 7.81 | 28.38, 39.38, 27.03 | 16.14, 26.48, 14.70 |
| 15 | 225 | 80 | 100 | 6 | 7.37, 10.27, 6.53 | 9.09, 13.61, 7.78 | 30.34, 40.78, 27.31 | 18.70, 27.03, 16.27 |
| 15 | 237 | 80 | 100 | 6 | 6.42, 9.69, 5.90 | 8.01, 12.69, 6.96 | 32.13, 41.46, 27.48 | 20.04, 27.15, 16.20 |
| 15 | 249 | 80 | 100 | 6 | 5.90, 9.71, 6.55 | 7.31, 13.22, 7.58 | 33.48, 44.16, 32.03 | 20.76, 30.07, 18.51 |
| 15 | 225 | 80 | 100 | 6 | 7.37, 10.27, 6.53 | 9.09, 13.61, 7.78 | 30.34, 40.78, 27.31 | 18.70, 27.03, 16.27 |
| 15 | 237 | 76 | 100 | 6 | 7.36, 10.20, 6.33 | 8.90, 13.36, 7.36 | 30.96, 40.97, 27.21 | 18.73, 26.83, 15.81 |
| 15 | 213 | 84 | 100 | 6 | 7.32, 10.35, 6.63 | 8.60, 14.01, 7.51 | 29.49, 40.15, 27.27 | 17.34, 27.18, 15.45 |
| 15 | 249 | 72 | 100 | 6 | 7.19, 10.27, 6.71 | 8.70, 13.57, 7.71 | 29.81, 41.08, 27.28 | 18.02, 27.15, 15.70 |
| 15 | 204 | 88 | 100 | 6 | 7.45, 10.48, 6.68 | 8.90, 13.92, 7.50 | 29.87, 40.71, 27.90 | 17.86, 27.04, 15.66 |
| 15 | 225 | 80 | 100 | 4 | 6.21 | 6.90 | 26.95 | 14.98 |
| 15 | 225 | 80 | 100 | 5 | 6.43 | 7.72 | 27.57 | 16.55 |
| 15 | 225 | 80 | 100 | 6 | 6.53 | 7.78 | 27.31 | 16.27 |
| 15 | 225 | 80 | 100 | 7 | 6.38 | 7.98 | 26.81 | 16.76 |
| 15 | 225 | 80 | 100 | 8 | 6.86 | 8.69 | 28.49 | 18.06 |
| 15 | 225 | 80 | 100 | 9 | 6.91 | 8.89 | 29.35 | 18.86 |
| 15 | 225 | 80 | 100 | 10 | 6.89 | 8.85 | 29.24 | 18.76 |
| 15 | 225 | 80 | 100 | 11 | 6.88 | 8.71 | 29.25 | 18.50 |
| 15 | 225 | 80 | 100 | 12 | 7.23 | 9.29 | 29.98 | 19.24 |
| 15 | 225 | 80 | 100 | 13 | 7.34 | 9.31 | 30.25 | 19.17 |
| 15 | 225 | 80 | 100 | 14 | 7.23 | 9.27 | 30.07 | 19.27 |
| 15 | 225 | 80 | 100 | 15 | 7.39 | 9.25 | 30.84 | 19.29 |
| 15 | 225 | 80 | 100 | 16 | 7.41 | 9.37 | 30.88 | 19.51 |
| 15 | 225 | 80 | 100 | 17 | 7.25 | 9.13 | 30.23 | 19.02 |
| 15 | 225 | 80 | 100 | 18 | 7.39 | 9.42 | 30.72 | 19.59 |
| 15 | 225 | 80 | 100 | 19 | 7.45 | 9.33 | 30.97 | 19.38 |
| 15 | 225 | 80 | 100 | 20 | 7.47 | 9.38 | 31.35 | 19.69 |
| 15 | 225 | 80 | 100 | 21 | 7.47 | 9.38 | 31.39 | 19.72 |
| 15 | 225 | 80 | 100 | 22 | 7.32 | 9.35 | 30.98 | 19.77 |
| 15 | 225 | 80 | 100 | 23 | 7.48 | 9.46 | 31.21 | 19.74 |
| 15 | 225 | 80 | 100 | 24 | 7.54 | 9.50 | 31.39 | 19.79 |
| 15 | 225 | 80 | 100 | 25 | 7.49 | 9.58 | 31.43 | 19.11 |
| 15 | 225 | 80 | 100 | 26 | 7.53 | 9.54 | 31.43 | 19.92 |
| 15 | 225 | 80 | 100 | 27 | 7.37 | 9.33 | 31.13 | 19.69 |
| 15 | 225 | 80 | 100 | 28 | 7.42 | 9.15 | 31.40 | 19.35 |
| 15 | 225 | 80 | 100 | 29 | 7.28 | 9.07 | 31.04 | 19.32 |
| 15 | 225 | 80 | 100 | 30 | 7.33 | 9.15 | 31.16 | 19.44 |
| 15 | 225 | 80 | 100 | 31 | 7.15 | 8.97 | 30.86 | 19.35 |
| 15 | 225 | 80 | 100 | 32 | 7.15 | 8.73 | 30.77 | 18.78 |
| 15 | 225 | 80 | 100 | 33 | 7.20 | 8.81 | 31.23 | 19.10 |
| 15 | 225 | 80 | 100 | 34 | 7.06 | 8.71 | 30.71 | 18.93 |
| 15 | 225 | 80 | 100 | 35 | 7.08 | 8.73 | 31.00 | 19.11 |
| 15 | 225 | 80 | 100 | 36 | 6.91 | 8.53 | 30.65 | 18.91 |
| 15 | 225 | 80 | 100 | 37 | 6.75 | 8.33 | 30.38 | 18.77 |
| 15 | 225 | 80 | 100 | 38 | 6.87 | 8.51 | 30.88 | 19.14 |

**Table C.** **Sensitivity analysis of the optimal discovery rates and specificities of statistically significant EWSs for the CHF-JPY exchange rates from 2008 to 2009.**

| $T_{0}$ | $R_{win}$ | $R_{ind}$ | $\sigma$ | $P$ | ${DR}_{10}$ (%) | ${DR}_{5}$ (%) | ${SP}_{10}$ (%) | ${SP}_{5}$ (%) |
| --- | --- | --- | --- | --- | --- | --- | --- | --- |
| 15 | 225 | 80 | 120 | 26 | 2.88, 7.44, 1.89 | 2.42, 8.47, 1.07 | 10.24, 30.87, 7.28 | 4.30, 17.58, 2.06 |
| 20 | 168 | 80 | 90 | 26 | 2.98, 7.52, 3.01 | 2.13, 7.66, 2.27 | 11.08, 30.77, 10.65 | 3.96, 15.67, 4.01 |
| 30 | 114 | 80 | 60 | 26 | 3.37, 7.10, 3.73 | 3.33, 7.89, 4.06 | 13.92, 30.20, 14.80 | 6.89, 16.80, 8.05 |
| 30 | 114 | 80 | 76 | 26 | 2.68, 6.91, 3.51 | 3.11, 7.68, 4.06 | 11.26, 30.32, 14.63 | 6.54, 16.83, 8.45 |
| 30 | 114 | 80 | 68 | 26 | 3.01, 7.06, 3.51 | 3.33, 7.89, 3.77 | 12.37, 30.33, 14.20 | 6.86, 16.95, 7.61 |
| 30 | 114 | 80 | 60 | 26 | 3.37, 7.10, 3.73 | 3.33, 7.89, 4.06 | 13.92, 30.20, 14.80 | 6.89, 16.80, 8.05 |
| 30 | 114 | 80 | 52 | 26 | 3.51, 6.95, 3.95 | 3.40, 7.82, 4.27 | 14.29, 28.49, 15.42 | 6.92, 16.02, 8.35 |
| 30 | 114 | 80 | 48 | 26 | 3.62, 7.10, 3.80 | 3.40, 7.97, 3.91 | 14.60, 28.45, 14.85 | 6.86, 15.97, 7.64 |
| 30 | 114 | 80 | 44 | 26 | 3.26, 7.06, 3.87 | 3.11, 7.75, 3.98 | 12.97, 27.74, 14.68 | 6.20, 15.22, 7.54 |
| 30 | 114 | 80 | 40 | 26 | 3.40, 7.35, 4.16 | 3.33, 8.18, 4.34 | 13.31, 27.73, 15.44 | 6.52, 15.44, 8.05 |
| 30 | 114 | 80 | 36 | 26 | 3.55, 7.46, 4.02 | 3.69, 8.40, 4.06 | 13.92, 27.21, 14.96 | 7.24, 15.32, 7.55 |
| 30 | 114 | 80 | 32 | 26 | 3.95, 7.53, 4.09 | 4.13, 8.40, 4.13 | 15.31, 27.15, 15.19 | 8.01, 15.14, 7.66 |
| 30 | 114 | 80 | 28 | 26 | 4.42, 7.57, 3.87 | 4.42, 8.69, 3.84 | 16.31, 27.04, 13.99 | 8.16, 15.52, 6.93 |
| 30 | 114 | 80 | 24 | 26 | 4.42, 7.49, 3.84 | 4.27, 8.69, 3.84 | 16.05, 26.61, 13.78 | 7.76, 15.42, 6.89 |
| 30 | 114 | 80 | 48 | 26 | 3.62, 7.10, 3.80 | 3.40, 7.97, 3.91 | 14.60, 28.45, 14.85 | 6.86, 15.97, 7.64 |
| 30 | 114 | 76 | 48 | 26 | 3.29, 7.57, 4.02 | 2.97, 8.25, 4.06 | 11.91, 28.71, 13.47 | 5.37, 15.66, 6.80 |
| 30 | 114 | 72 | 48 | 26 | 3.48, 8.00, 4.45 | 3.40, 8.69, 4.78 | 11.29, 29.00, 12.93 | 5.53, 15.75, 6.94 |
| 30 | 114 | 84 | 48 | 26 | 3.29, 7.20, 3.84 | 3.11, 8.40, 4.06 | 15.14, 29.70, 17.01 | 7.15, 17.31, 8.99 |
| 30 | 114 | 88 | 48 | 26 | 3.26, 6.63, 3.80 | 3.26, 7.46, 3.98 | 16.57, 27.19, 19.20 | 8.29, 15.30, 10.05 |
| 30 | 78 | 84 | 48 | 26 | 3.42, 8.22, 4.56 | 3.37, 8.42, 4.41 | 8.98, 28.21, 11.48 | 4.42, 14.44, 5.55 |
| 30 | 90 | 84 | 48 | 26 | 3.46, 8.63, 4.32 | 3.37, 9.83, 3.83 | 9.91, 28.30, 12.11 | 4.83, 16.12, 5.37 |
| 30 | 102 | 84 | 48 | 26 | 3.34, 8.03, 3.92 | 2.98, 8.88, 3.89 | 11.31, 29.70, 12.64 | 5.05, 16.41, 6.27 |
| 30 | 114 | 84 | 48 | 26 | 3.29, 7.20, 3.84 | 3.11, 8.40, 4.06 | 15.14, 29.70, 17.01 | 7.15, 17.31, 8.99 |
| 30 | 126 | 84 | 48 | 26 | 3.28, 7.16, 4.72 | 3.36, 7.77, 4.96 | 18.94, 29.59, 26.28 | 9.70, 16.03, 13.81 |
| 30 | 138 | 84 | 48 | 26 | 3.68, 5.96, 5.00 | 3.68, 6.93, 5.35 | 18.92, 28.22, 24.52 | 9.46, 16.39, 13.12 |
| 30 | 150 | 84 | 48 | 26 | 5.38, 7.91, 6.24 | 5.15, 9.82, 6.58 | 22.03, 33.54, 25.19 | 10.53, 20.81, 13.27 |
| 30 | 162 | 84 | 48 | 26 | 5.35, 9.06, 6.48 | 4.84, 10.61, 5.77 | 19.30, 33.21, 21.36 | 8.72, 19.43, 9.49 |
| 30 | 174 | 84 | 48 | 26 | 5.69, 9.51, 6.19 | 4.31, 10.73, 5.31 | 18.90, 33.73, 19.55 | 7.16, 19.02, 8.38 |
| 30 | 132 | 96 | 48 | 26 | 5.49, 8.51, 6.92 | 4.78, 10.23, 6.71 | 22.09, 34.29, 26.61 | 9.61, 20.61, 12.90 |
| 30 | 138 | 92 | 48 | 26 | 5.30, 8.33, 6.18 | 4.65, 10.44, 5.96 | 20.65, 34.61, 23.94 | 9.04, 21.68, 11.55 |
| 30 | 144 | 88 | 48 | 26 | 5.54, 8.23, 6.59 | 4.67, 10.16, 6.22 | 22.70, 34.16, 26.72 | 9.59, 21.06, 12.62 |
| 30 | 150 | 84 | 48 | 26 | 5.38, 7.91, 6.24 | 5.15, 9.82, 6.58 | 22.03, 33.54, 25.19 | 10.53, 20.81, 13.27 |
| 30 | 159 | 80 | 48 | 26 | 5.51, 7.47, 6.16 | 4.65, 8.79, 6.26 | 22.71, 32.82, 25.90 | 9.58, 19.29, 13.16 |
| 30 | 165 | 76 | 48 | 26 | 5.45, 7.76, 5.92 | 4.61, 9.12, 5.87 | 22.66, 34.91, 24.41 | 9.59, 20.52, 12.10 |
| 30 | 174 | 72 | 48 | 26 | 5.53, 7.74, 6.14 | 4.09, 9.18, 5.53 | 23.64, 35.09, 25.75 | 8.75, 20.80, 11.60 |
| 30 | 150 | 84 | 48 | 20 | 5.48 | 5.62 | 22.95 | 11.78 |
| 30 | 150 | 84 | 48 | 21 | 5.57 | 5.53 | 22.85 | 11.33 |
| 30 | 150 | 84 | 48 | 22 | 5.86 | 6.01 | 23.25 | 11.91 |
| 30 | 150 | 84 | 48 | 23 | 6.00 | 6.01 | 23.68 | 11.84 |
| 30 | 150 | 84 | 48 | 24 | 6.53 | 6.77 | 25.46 | 13.20 |
| 30 | 150 | 84 | 48 | 25 | 6.53 | 6.86 | 24.91 | 13.09 |
| 30 | 150 | 84 | 48 | 26 | 6.24 | 6.58 | 25.19 | 13.27 |
| 30 | 150 | 84 | 48 | 27 | 5.91 | 6.39 | 25.05 | 13.53 |
| 30 | 150 | 84 | 48 | 28 | 5.53 | 6.01 | 23.06 | 12.52 |
| 30 | 150 | 84 | 48 | 29 | 5.10 | 5.24 | 20.94 | 10.76 |
| 30 | 150 | 84 | 48 | 30 | 4.43 | 4.48 | 18.98 | 9.59 |
| 30 | 150 | 84 | 48 | 31 | 4.29 | 4.29 | 18.48 | 9.24 |
| 30 | 150 | 84 | 48 | 32 | 4.38 | 4.67 | 18.36 | 9.78 |

**Table D.** **Average percentage changes of DR5 and SP5 for 1% changes of the parameters around the optimal values, of EWSs from AC(1), Var, and LFPS, for all three data sets. Derived from Tables A, B, and C.**

| Data set | Parameter | ${DR}_{5}$ change percentage | ${SP}_{5}$ change percentage |
| --- | --- | --- | --- |
| AUD-JPY  1996 to 2004 | $T_{0}$ | 0.1824 0.6932 0.0656 | 0.4368 0.7810 0.1802 |
|  | $\sigma$ | 0.1584 0.0931 0.1863 | 0.2642 0.0571 0.1458 |
|  | $R_{ind}$ | 0.4413 1.4000 0.7020 | 0.5053 1.1266 0.6500 |
|  | $R_{win}$ | 0.8353 1.0963 0.4541 | 0.6102 0.9172 0.2430 |
|  | $R_{ind}$ and $R_{win}$ | 0.3940 0.3151 0.3266 | 0.1683 0.3467 0.4857 |
|  | *P* | 0.491 | 0.4571 |
| AUD-JPY  2005 to 2010 | $T_{0}$ | 0.2244 0.0525 0.8017 | 0.1341 0.0186 0.8221 |
|  | $\sigma$ | 0.1870 0.0370 0.1800 | 0.1845 0.0385 0.0430 |
|  | $R_{ind}$ | 1.9030 1.3510 1.1950 | 1.1500 0.2700 0.5780 |
|  | $R_{win}$ | 1.3518 1.5300 1.0253 | 1.9568 0.2318 0.9456 |
|  | $R_{ind}$ and $R_{win}$ | 0.7480 0.4780 0.8870 | 0.7430 0.1290 0.7870 |
|  | *P* | 0.3402 | 0.6779 |
| CHF-JPY  2008 to 2009 | $T_{0}$ | 1.0813 0.0876 1.3228 | 1.2760 0.2019 1.5059 |
|  | $\sigma$ | 0.5120 0.2785 0.6603 | 0.6297 0.3007 0.6362 |
|  | $R_{ind}$ | 1.4853 1.7132 0.5946 | 2.1008 2.0326 2.8162 |
|  | $R_{win}$ | 2.1600 2.3418 1.9375 | 1.7093 1.7418 1.8512 |
|  | $R_{ind}$ and $R_{win}$ | 1.9989 1.4654 1.0851 | 1.8856 0.8929 0.6019 |
|  | *P* | 1.506 | 1.3345 |
